# Gradient-Based Time-Sequential Potential Field Method for Path Planning in Infrastructure-Based Cooperative Driving Automation

**DOI:** 10.3390/s26072163

**Published:** 2026-03-31

**Authors:** Jakyung Ko, Inchul Yang

**Affiliations:** 1 Department of Civil and Environmental Engineering, University of Science and Technology, Daejeon 34113, Republic of Korea; rhruf55@naver.com; 2 Department of Highway and Transportation, Korea Institute of Civil Engineering and Building Technology, Goyang-si 10223, Republic of Korea

**Keywords:** automated driving, path planning, potential field, cooperative automated driving, infrastructure-based cooperative driving

## Abstract

This study proposes a GTS-PF (Gradient-based Time-Sequential Potential Field)-based path generation method for real-time reference-path planning at infrastructure-based RSUs in cooperative driving automation environments. Conventional path planning approaches exhibit limitations in computational lightweight characteristics or responsiveness to dynamic environments, which restrict their suitability for negotiation-oriented reference-path generation and dissemination. To address these limitations, the proposed GTS-PF framework interprets the prediction time horizon as a sequence of updated temporal layers, enabling adaptive responses to dynamic obstacle variations. The method is formulated based on potential field principles to allow efficient computation while incorporating diverse interaction effects. A key feature of the proposed approach is the separation of direction planning and speed planning for obstacle avoidance, wherein a candidate acceleration set is generated based on future risk evaluation. Simulation results in an overtaking scenario involving a low-speed preceding vehicle demonstrate that the proposed method satisfies predefined safety and path-quality criteria. Moreover, the computation time was reduced by 81% compared to the baseline method, confirming computational lightweight feasibility for RSU-level implementation and demonstrating applicability in infrastructure-led cooperative driving automation.

## 1. Introduction

Cooperative driving automation has been proposed as an approach to enhance conventional autonomous driving through path sharing and V2X (Vehicle-to-Everything) cooperation among autonomous vehicles. Early cooperative driving was limited to the exchange of information collected by individual vehicles; however, advances in vehicle-to-vehicle communication, such as V2V and V2I, have enabled real-time sharing and agreement on vehicle states, positions, velocities, and driving intentions [1,2]. As a result, vehicles can move beyond independently determining future driving behaviors and instead pursue safe and efficient motion through maneuver coordination [3].

In cooperative driving environments, road infrastructure supports information collection while also mediating inefficiencies and conflicts that may arise when individual vehicles pursue their own interests during coordination processes. Through this role, infrastructure can allocate optimized paths and actions from a system-level traffic perspective. Accordingly, infrastructure plays a leading role in facilitating vehicle cooperation and supporting autonomous vehicles on the road [4,5]. In particular, the ability to rapidly derive executable paths for individual vehicles in response to real-time dynamic changes can substantially support maneuver allocation and coordination.

Paths provided at the infrastructure level are required to satisfy several conditions. They must be capable of responding to dynamic environmental changes and be computationally lightweight to enable real-time operation on Road-Side Units (RSUs) [6]. Furthermore, while accounting for positional and intentional relationships among multiple vehicles within the structural constraints of road environments, an appropriate level of executability that real vehicles can follow is required.

Accordingly, the objective of this study is to propose an executable path generation method that incorporates temporal information for infrastructure-based cooperative autonomous driving environments, while seeking a balance between path quality and computational efficiency. To this end, this study focuses on potential-field-based approaches. Potential field methods represent various environment-dependent factors as resultant forces, allowing efficient path generation in complex driving scenarios and addressing practical infrastructure requirements related to lightweight computation and multi-factor consideration.

To apply potential field methods to infrastructure-based cooperative driving environments, this study proposes a Gradient-based Time-Sequential Potential Field (GTS-PF) path generation method that updates and reflects temporal information in a layered manner. To address the limitations of conventional potential-field-based path planning methods, which typically assume static environments or fixed time axes and thus exhibit limited real-time adaptability and executability, the proposed method dynamically updates potential fields based on predicted driving situations in road space while sequentially assigning temporal information to path points. Through this structure, the proposed method is suitable for real-time infrastructure-assisted cooperative driving, as it can respond promptly to environmental changes while maintaining an appropriate level of executability under lightweight computation.

Consequently, the contributions of this study include the development of a lightweight dynamic path planning method applicable to infrastructure-based cooperative autonomous driving environments, improvements in temporal information handling, and the achievement of safety and dynamic path quality.

To describe the proposed method, Section 2 presents a literature survey reviewing prior studies on potential fields in road traffic domains and potential-field-based path generation. Section 3 presents the path generation principles of the proposed method. Section 4 evaluates the performance of the proposed method through simulations and compares it with existing approaches, including Gradient-based Time-Extended Potential Field (GT-PF), a potential-field-based path planning method with extended spatiotemporal representation, for efficiency assessment. The comparison method explores paths by following potential field gradients in a three-dimensional space with an extended time axis, which enables simultaneous consideration of spatial and temporal information but may present limitations in infrastructure environments due to prediction uncertainty and computational burden. In this study, the two methods are compared and analyzed in specific scenarios to verify the feasibility of generating paths that satisfy requirements for real-time performance, driving stability, and executability. Section 5 discusses the limitations, future research directions, practicality, and extensibility of this study and presents concluding remarks.

## 2. Related Works and Research Trends

### 2.1. Potential Field Applications in the Transportation Domain

**Figure 1:**
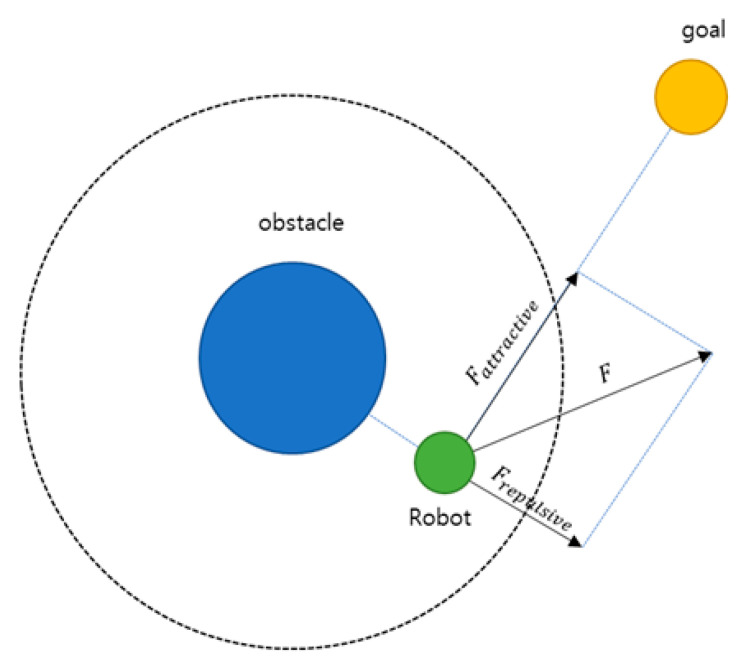
Attractive force and repulsive force in APF [21].

Potential field theory originated from the Artificial Potential Field (APF) model proposed by [7]. As illustrated in Figure 1, the APF combines an attractive force that guides a mobile agent toward a goal with a repulsive force that prevents collisions with obstacles, thereby enabling path generation by simplifying interactions between the agent and the environment on a two-dimensional plane. Through this approach, obstacle avoidance and goal reaching can be achieved simultaneously in a relatively concise manner, even in complex environments.

Subsequently, various extension studies have been conducted in the transportation domain to overcome the limitations of planar APF applications and to incorporate the specific characteristics of road environments into potential field theory. Road lanes, boundaries, structures, and vehicles were represented as attractive and repulsive components of potential fields, enabling vehicles to maintain lanes and prevent road departure or collisions [8,9,10,11,12]. In particular, ref.[11] introduced the concept of a driving safety field for road environments and proposed a model that jointly considers infrastructure and vehicle-related elements. To account for interactions with dynamic obstacles such as moving vehicles, ref.[13] introduced the Yukawa potential into vehicle potential formulations, allowing the distribution of repulsive risk to vary according to velocity and acceleration, and explored predictive modeling approaches for complex road scenarios. Reference [14] formulated potential fields from the perspective of driver-perceived risk, while [15] further enhanced dynamic vehicle potential field models by incorporating acceleration, safety distance, and velocity variables, thereby extending their applicability to vehicle interaction and driver behavior analysis.

In addition to path planning, potential-field-based risk models in the transportation domain have also been applied to driver behavior analysis and safety assessment. Reference [16] developed models that reflect drivers’ perceived risk to better capture cognitive and reaction behaviors. Reference [17] proposed models that include pedestrians, while [18] constructed models that predictively represent latent and unperceived objects within the field, thereby broadening the range of factors considered in risk evaluation. Reference [19] employed potential fields to perform continuous risk estimation, visualization, and safety metric generation, and [20] applied potential fields to real-time risk assessment in multi-CAV (Connected Automated Vehicle) environments.

Overall, potential field theory in the transportation domain has evolved from APF-based collision-free path generation on planar surfaces toward approaches that more realistically represent road structures, vehicles, pedestrians, and dynamic entities, while enabling the evaluation of vehicle interactions and associated risks. More recently, its application scope has further expanded to local path generation for real-time cooperative driving and to risk assessment and safety systems applicable to multi-CAV environments.

#### 2.1.1. Vehicle Potential Formulation

Because the influence exerted by vehicles differs from that of general obstacles, a vehicle-specific potential formulation is constructed in this study. The proposed vehicle-based potential function is designed to capture both the dynamic characteristics of vehicles and the directional driving characteristics imposed by lane-based road structures. To represent distance-dependent attenuation effects, the formulation is adapted from the Yukawa potential, which has been employed in prior studies on leading vehicle potential modeling [21]. Furthermore, vehicle velocity and acceleration are incorporated to asymmetrically represent region-specific risk characteristics.

(1) $$U_{vehicle}=\frac{k_{v}\cdot v+\tau }{s^{\prime }+\epsilon_{1}}\times \exp\left(\frac{1}{v+\epsilon_{2}}\left(\frac{a\left(x-x_{0}\right)}{\left|a\right|+\epsilon_{3}}-s^{\prime }\right)\right)$$

In this formulation, *s*′ represents the virtual distance between the assumed vehicle center ($x_{0}$, $y_{0}$) and the location at which the potential is evaluated. As the virtual distance increases, the resulting potential decreases. The coefficient $k_{v}$ scales the magnitude of the potential according to the vehicle velocity v, while τ determines the influence of a stationary vehicle and the baseline safety distance. Higher vehicle speeds result in stronger and more spatially extended potential influence. The acceleration term a represents future driving intent and determines the longitudinal extent of potential influence in the forward and backward directions. The coefficients used in this formulation were selected based on prior studies on vehicle potential modeling and spatiotemporal risk representation [21,22].

#### 2.1.2. Virtual Distance and Lateral Interaction Effect

The virtual distance $s^{\prime }$ is computed by modifying the distance ratio to account for differences in longitudinal and lateral influence between the evaluation point and the vehicle. Equation (2) describes the transformation from the standard Euclidean distance s to the virtual distance $s^{\prime }$, which reflects lateral interaction effects.

(2) $$\begin{array}{c} s=\sqrt{{\left(x-x_{0}\right)}^{2}+{\left(y-y_{0}\right)}^{2}}\\ s^{\prime }=\sqrt{{c_{1}^{2}(x-x_{0})}^{2}+{c_{2}^{2}(y-y_{0})}^{2}} \end{array}$$

**Figure 2:**
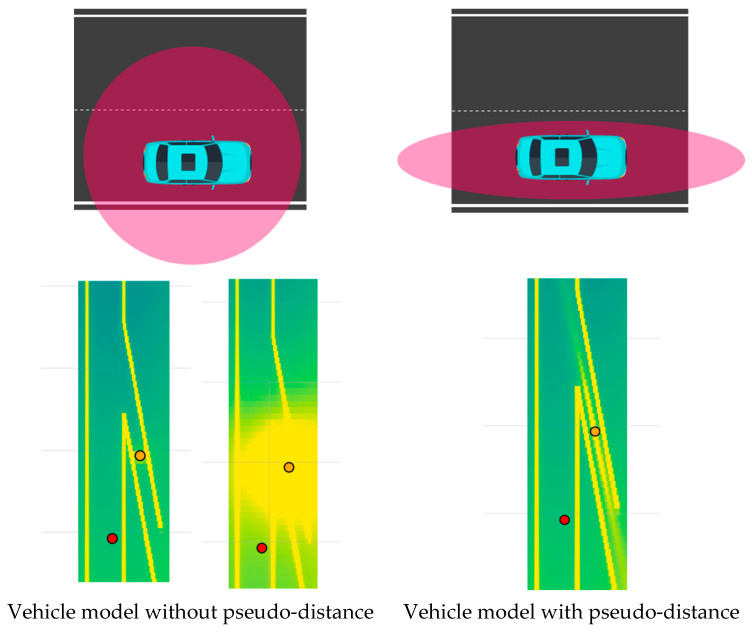
Effect of Pseudo-Distance on vehicle model shape.

In general, vehicles exhibit greater freedom of motion in the longitudinal direction than in the lateral direction. This characteristic originates from both vehicle dynamics and the lane-separated structure of road environments. In lane-based driving scenarios, vehicles traveling in parallel lanes are typically not perceived as threatening. Accordingly, lateral influence is reduced so that vehicles located in adjacent lanes are not significantly affected, as illustrated in Figure 2. According to the findings reported, the potential influence exerted by a vehicle within a 30 m longitudinal range is comparable to that within a 2 m lateral range [13]. Based on this observation, parameters $c_{1}$ and $c_{2}$ are applied to adjust the scale of lateral influence when deriving the virtual distance $s^{\prime }$.

The scaling parameters $c_{1}$ and $c_{2}$ are determined based on prior studies [13], where the anisotropic characteristics of vehicle interaction were analyzed. In this study, these parameters are applied in a fixed manner to reflect the relative importance of longitudinal and lateral interactions. While this formulation captures the essential interaction characteristics in lane-based environments, adaptive tuning of these parameters depending on driving context (e.g., lane change or road geometry) may further improve performance and is left for future work.

### 2.2. Path Generation Using Potential Fields

The fundamental principle of potential-field-based path generation is to follow the gradient of the composite potential field defined over the entire space, that is, to move in the direction of the steepest potential decrease in order to identify a low-risk path. This approach allows various environmental elements, including obstacles, road boundaries, and destinations, to be simultaneously incorporated through a combination of attractive and repulsive forces. As a result, at a given time instant, the vehicle can immediately determine a movement direction that minimizes risk, enabling relatively simple real-time path generation even in complex environments. However, vehicles may become trapped in local minima caused by obstacles or boundaries, which can interrupt path generation or prevent convergence to an optimal path. Moreover, because decision-making is typically limited to instantaneous path determination, such approaches have difficulty capturing time-varying dynamics, including moving obstacles and leading vehicles, as well as incorporating vehicle dynamic constraints and environmental changes at traversal time. To address these limitations, numerous extensions and hybrid approaches have been explored in the literature.

#### 2.2.1. Hybrid Path Generation Using Potential Fields

One line of research has focused on combining potential fields with sampling-based path planning methods. References [23,24] integrated potential fields into the cost formulation of the A* algorithm to increase costs in obstacle-adjacent regions, thereby encouraging avoidance behavior. Reference [25] further combined the A*–APF framework with Bézier-curve-based smoothing to enhance the quality of vehicle and pedestrian avoidance paths. In the context of sampling-based planners such as RRT and RRT*, the authors of [26,27,28] introduced potential field guidance to bias path expansion toward goal attraction and obstacle repulsion directions, mitigating the limitations of random sampling and alleviating issues such as local minima, oscillatory behavior, and inefficient paths.

In parallel, optimal-control-based approaches have been integrated with potential fields to enhance real-time performance and execution feasibility. Rasekhipour et al. (2016) [29] combined model predictive control (MPC) with potential-field-based planning by incorporating potential values, path-tracking errors, and velocity, acceleration, and steering-rate variations into the cost function, while explicitly accounting for vehicle dynamics to jointly optimize path, speed, and steering. Related studies further explored and validated diverse combinations of optimal control and potential fields for real-time obstacle avoidance, cooperative driving, and operation under various road curvature conditions using two- and four-wheel vehicle models [30,31,32,33,34,35,36].

#### 2.2.2. Dynamic Path Generation Using Potential Fields

Another research direction assigns realistic velocity profiles to potential-field-based paths. Reference [37] modeled potential-field-generated paths on an S–T (space–time) graph to avoid dynamic obstacle occupancy regions and proposed a speed-matching strategy that jointly considers safety distance, desired speed, acceleration, and jerk minimization. To overcome the limitations of static-time potential field application, several studies have extended potential fields into three-dimensional spatiotemporal domains. Reference [38] proposed the Spatial–Temporal Risk Field (STRF) model, in which path exploration is conducted using RRT within the (x, y, t) space. These studies build upon the core structure of potential-field-based planning while incorporating methodological extensions to meet practical deployment requirements. Recent studies have further extended spatiotemporal risk field concepts using data-driven formulations, enabling adaptive risk representation under complex traffic conditions [39]. In addition, recent learning-based approaches have explored end-to-end driving frameworks that implicitly capture environmental risk through learned representations, further expanding the applicability of field-based interpretations [40].

#### 2.2.3. Dynamic Path Generation Using GT-PF

As prior work aimed at developing a lightweight path planning approach for infrastructure-based cooperative driving environments, a GT-PF-based method was introduced [22]. This approach integrates time into the spatial coordinate system via scale transformation and represents spatiotemporal environmental changes and vehicle trajectories within a continuous three-dimensional space. Vehicles treat temporal progression as spatial displacement during planning. Quantitative analysis revealed several limitations: while collision avoidance at specific time instances was achieved, concatenation of straight unit time vectors representing constant velocity led to discontinuous velocity transitions and excessive acceleration. In addition, flexible time update intervals caused small adjustments to accumulate over short durations, resulting in unstable motion. Furthermore, because GT-PF relies on continuously evolving spatiotemporal potential fields, it is difficult to locally update the field when actual environmental conditions deviate from prior predictions. The vehicle potential model, which embeds future acceleration and driving intent at a single time instance, also results in redundant future information and excessive intent projection in time-extended environments.

Accordingly, limitations arise when applying conventional potential-field-based planning methods to real cooperative driving scenarios. These limitations primarily concern computational efficiency and path quality, motivating the development of a new path planning approach.

## 3. Proposed Method

### 3.1. Overview

To overcome the limitations of existing potential-field-based path planning approaches, this study retains the concept of generating dynamic paths for cooperative autonomous driving environments using potential fields, while improving the manner in which temporal variation is handled. Consistent with prior work, path exploration over a predicted time horizon is performed without introducing optimization, inverse computation, or iterative refinement at each step. Instead of integrating the time axis into the spatial coordinate system, space and time are treated as separate dimensions. Accordingly, time is discretized into fixed intervals, and a GTS-PF (Gradient-based Time-Sequential Potential Field) path planning method is proposed, in which independently updated potential fields at each time step are sequentially connected.

In GTS-PF, the progression of time is represented using layer units, and at each layer a potential distribution is generated to reflect changes in the driving environment and vehicle position over a unit time interval. The vehicle determines its movement direction by following the potential gradient of the current layer, while the movement magnitude is determined using predicted information from the subsequent layer. Rather than assuming constant-velocity motion, the movement magnitude determined at each layer is realized through acceleration control, allowing continuous velocity variation until the target movement magnitude is reached. This process is repeated until the destination is reached, and the paths generated at individual layers are connected to form the final path.

Under this framework, GTS-PF evaluates only a single time layer at each planning step, while short-term future influences up to the next update are referenced through velocity and acceleration terms embedded within the potential function to account for potential future risks. Consequently, each layer can be treated as a simplified path planning problem analogous to a static potential field defined at a single time instant. Moreover, because movement direction and movement magnitude are determined sequentially rather than jointly at each layer, the number of candidate cases to be explored is reduced compared to coupled optimization-based approaches. The fixed time update interval further prevents the accumulation of dense fluctuations over short durations, enabling relatively simple and stable path exploration.

Following this sequential planning structure, GTS-PF does not require re-planning, backtracking, or repeated optimization, and decisions at each time step are performed independently based on the position and velocity determined at the previous step. The resulting modular structure, in which each unit of the planning flow is separated, allows local components—such as risk evaluation methods, movement magnitude determination, and layer-specific decision criteria—to be replaced without modifying the overall framework. As a result, GTS-PF is designed to achieve path generation with lightweight computation by clearly defining and constraining the role of each decision stage.

Ultimately, rather than exploring a single potential space defined over a continuous time axis, GTS-PF divides time into discrete prediction units, independently computes the environment at each time step, and incrementally accumulates and connects the results. This structure enables localized updates in response to environmental changes and allows only the affected segments to be recalculated when predicted information deviates from actual conditions. In doing so, GTS-PF preserves the dynamic extensibility of potential-field-based planning through time and velocity handling inherent in GT-PF, while simultaneously ensuring real-time capability and adaptability, making it suitable and practical for planning lightweight reference paths in infrastructure-led cooperative autonomous driving.

### 3.2. Time-Sequential Path Planning Structure

GTS-PF is structured by decomposing the space at each time step into layer units along the temporal axis within the predicted spatiotemporal range. At each time step, decision-making is performed based on the potential gradient of the corresponding layer and predicted risks at future reachable positions, while a spatiotemporally consistent path is generated by sequentially connecting the results over the prediction horizon.

A key characteristic of path planning with GTS-PF is the separation of movement direction and speed determination. Movement direction is determined exclusively using the potential field gradient of the current time layer, selecting the safest and most goal-consistent direction with respect to that layer. This enables immediate directional responsiveness based on the vehicle’s position and surrounding environment, ensuring short-term collision avoidance and driving stability. In contrast, movement speed is determined by cumulatively considering future variations rather than relying on a single time instant. Along the direction determined at the current position, vehicle trajectories over a future interval are predicted for each candidate acceleration, and the associated risk variations are evaluated during progression, after field updates, and at time points where the driving tendency is maintained. Through this accumulation of future information, the system avoids myopic decision-making and aims to achieve long-term safety and efficiency.

In the previous section, the principle of gradient-descent-based direction determination—where a vehicle moves along the potential field gradient at a given time instant—was described. The GTS-PF path exploration method directly adopts this principle; however, because the potential field is recalculated at every step in a dynamic environment, the vehicle heading is always determined based on the updated field. Thus, directional decisions at each time step rely solely on the current layer’s potential field, ensuring responsiveness to immediate spatial risk factors such as road boundaries, lane centerlines, and leading vehicles.

**Figure 3:**
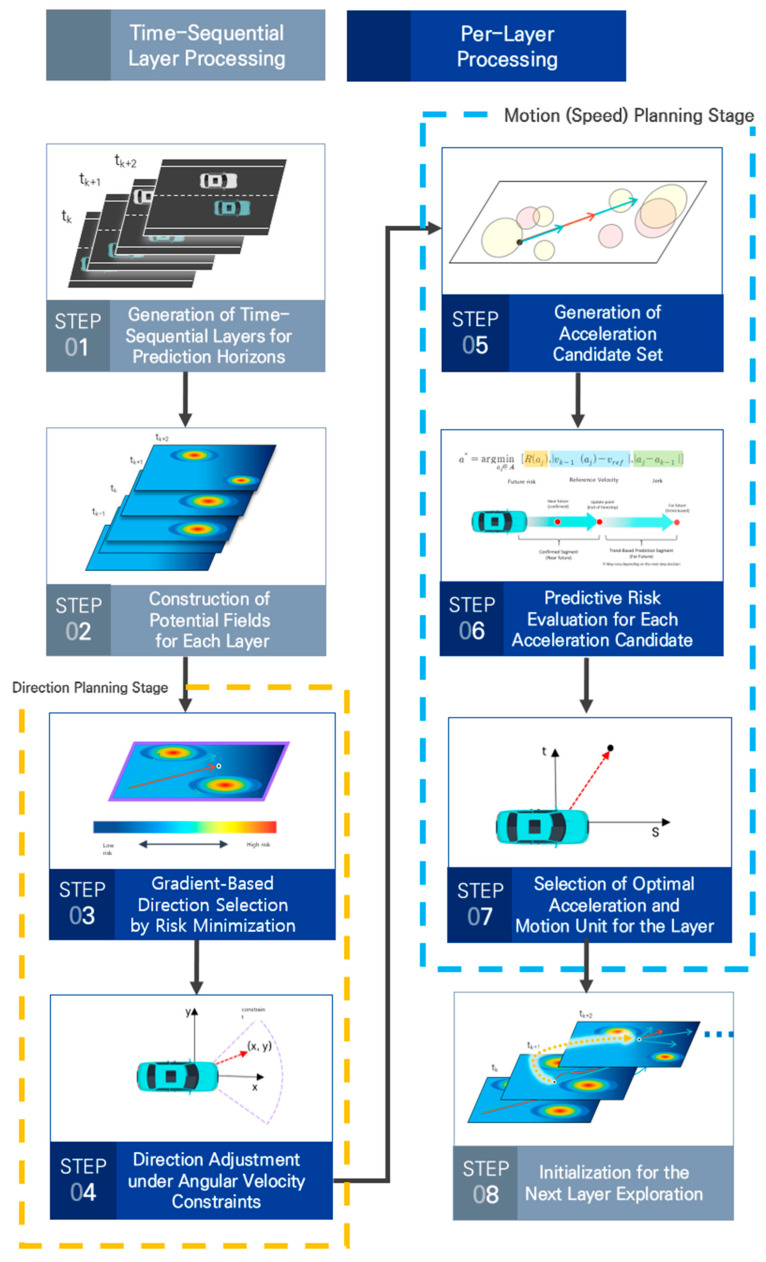
Overall flowchart of the GTS-PF method.

In contrast to direction determination, speed determination cannot be sufficiently addressed using information from the current time step alone. As the vehicle moves over time, continuous changes occur in leading vehicle motion, traffic flow, and the vehicle’s own position. Accordingly, speed is determined using a method that predicts changes across future layers up to reachable positions and derives a stable outcome by comprehensively considering these predictions. Figure 3 illustrates this process.

### 3.3. Potential-Gradient-Based Direction Planning

In potential-field-based path planning, the movement direction and movement magnitude of a vehicle are determined using a gradient descent approach. In practice, analytical differentiation of the potential function is often intractable; therefore, numerical differentiation based on a central difference scheme is employed. Given a target position for gradient computation, the gradient is approximated by evaluating the potential difference in both directions with a small perturbation h, as expressed in Equation (3).

(3) $$\nabla U(p_{k})\approx \frac{U\left(p_{k}+h\right)-U(p_{k}-h)}{2h}$$

Here, the step size h corresponds to the spatial resolution of the discretized grid used for potential evaluation. This formulation computes the gradient of the potential field directly, rather than applying differentiation to an already computed gradient.

While gradient descent along the direction of decreasing potential does not guarantee convergence to a globally optimal path, it enables rapid determination of the movement direction based solely on local information. At each layer update, the vehicle responds in real time to the provided environmental information. Consequently, when the potential field and the resulting gradients are appropriately formulated, diverse environmental risk factors—including complex road structures, dynamic obstacles, and leading vehicles—can be effectively avoided, allowing the vehicle to follow an efficient path toward the destination. The search region is restricted to a limited area ahead of the vehicle, and steering adjustments are constrained within an allowable angular range relative to the previous heading to prevent abrupt directional changes.

### 3.4. Acceleration-Based Motion Unit Planning

#### 3.4.1. Acceleration Candidate Set Definition

In the proposed path exploration framework, speed control is performed using an acceleration-based approach. Compared to direct speed control, acceleration control naturally ensures continuity and smoothness of motion. In particular, physical forces acting on real vehicles and friction-related effects are directly associated with acceleration, making acceleration-centered control more consistent with realistic driving behavior than direct speed manipulation. According to [41], vehicle control designs based on longitudinal acceleration can more closely replicate human driving behavior. Accordingly, in practical autonomous driving systems such as Adaptive Cruise Control (ACC) and optimal control algorithms, acceleration and deceleration are computed to generate smooth vehicle motion, thereby achieving movement similar to human driving behavior and ensuring ride comfort and smoothness. Furthermore, acceleration enables clearer prediction of future speed trends and provides a stable basis for risk avoidance and desired speed tracking.

In contrast, direct speed control simplifies distance computation by directly calculating a target speed at the current time and applying it as a speed command. However, since speed is independently controlled at each instant, it is difficult to ensure continuity in speed variation or acceleration. Such limitations may result in abrupt acceleration or deceleration and dynamic instability. Therefore, in this study, acceleration is selected as the control variable for path exploration instead of speed, and acceleration candidates are generated and evaluated at each step.

To implement acceleration control, an acceleration candidate set is constructed at each time step, centered on the acceleration value from the previous step and bounded within an allowable range. This range is defined by considering vehicle acceleration limits and jerk constraints, and candidates are generated by sampling the allowable acceleration variation range at a fixed resolution. Because the resolution interval determines both computational cost and sensitivity of speed adjustment, appropriate selection of this interval is important. This interval is defined as the acceleration resolution Δa, and Equation (4) defines the resulting acceleration candidate set.

(4) $$A_{k}=\left\{a_{j}|a_{min}{\leq a}_{j}{\leq a}_{max},a_{j}=a_{min}+(j-1)\Delta a\right\}$$

The number of acceleration candidates is given by $n=\frac{a_{max}-a_{min}}{\Delta a}$. To evaluate candidates within this set, it is necessary not only to consider the current state but also to predict the evolution of future states under each candidate acceleration. Through this evaluation process, an acceleration candidate whose future state remains safe and whose rate of change is appropriate is selected.

#### 3.4.2. Future-State Risk Evaluation

In this study, the duration from one time step to the next is defined as a time unit dt, and 3 types of prediction time points are specified based on this unit. The set of future prediction time points used for decision-making with respect to the current time is defined as shown in Equation (5), including the interval from the current time to the k-th step, the k-th step itself, and the prediction time beyond the k-th step.

(5) $$T_{k}=\tau_{pre(k+1)}\cup \tau_{k+1}\cup \tau_{post(k+1)}$$

Let $t_{k}$ denote the current update time, dt the interval until the next update, and $p_{k}$ the current vehicle position. Given the current velocity $v_{k}$, a set of acceleration candidates $a_{j}$ is defined to determine the acceleration $a_{k}$. For a future elapsed time τ from the current time, each acceleration candidate yields a different predicted position. The scalar distance s represents the traveled distance under the assumption of constant acceleration until τ. For each elapsed time, a predicted position $p(t_{k}+\tau ;a_{j})$ is generated, and the environmental potential at that position is evaluated.

**Figure 4:**
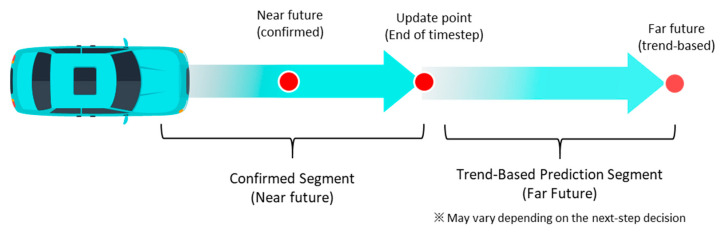
Future prediction for acceleration candidates.

As illustrated in Figure 4, the selected τ values can be classified into three regions. First, the movement interval from the current time to the next update necessarily occurs; therefore, its associated risk is assigned a higher weight. Second, the prediction point immediately after the update, corresponding to $t_{k+1}$, represents the position reached under the assumption that the current acceleration is maintained throughout the update interval and is strongly weighted as a confirmed segment. The consistency between predicted and reached positions may also serve as a criterion for assessing whether the vehicle motion state approaches the desired speed. Third, longer-term future points are predicted based on trend continuation, assuming similar behavior after the elapsed time, i.e., maintaining acceleration and speed change trends. Because these points may deviate depending on subsequent decisions, exponentially decaying weights are applied as the temporal distance increases. When an acceleration candidate is selected, the approximated travel distance and future position based on a constant-acceleration motion model are defined as shown in Equation (6).

(6) $$\begin{array}{c} s\left(\tau ,a_{j}\right)=v_{k-1}\;\tau +\frac{1}{2}a_{j}\tau^{2}\\ p\left(\tau ,a_{j}\right)=p_{k}+s(\tau ,a_{j}){\hat{d}}_{k} \end{array}$$

By jointly considering these 3 types of time points, the proposed system ensures immediate safety while further enhancing long-term stability. In principle, the most ideal representation of risk would be obtained by integrating the potential over the entire continuous time horizon. However, in real-time path planning, computing integrals over extensive prediction intervals for all acceleration candidates at every update step would impose excessive computational burden and is impractical for rapid path generation. Therefore, this study adopts a representative-point-based approach. A limited number of representative points are extracted from the three previously defined time-point categories and collectively used for risk evaluation. Different importance weights are assigned to these points: near-future segments with determined motion are weighted more heavily, whereas long-term trend-based prediction segments are reflected with attenuated weights. This approach enables reasonable risk evaluation that accounts for future variations while maintaining computational efficiency in real-time systems. Accordingly, risk values at selected future time points are incorporated as defined in Equation (7).

(7) $$U\left(\tau ,a_{j}\right)=U(p_{k}\left(\tau ,a_{j}\right),t_{k}+\tau )$$

Even if the position reached by selecting an acceleration candidate $a_{j}$ is currently safe, it does not guarantee safety at the future arrival time. That position may become occupied by other risk factors, or conversely, a location that is currently hazardous may become unoccupied at a future time. If a location that is highly safe at the current time exhibits significant risk at a future time, simple averaging may yield a moderate risk value, potentially leading to an erroneous decision that entry is permissible. In this context, aggregating risks across multiple time points using simple summation or averaging may obscure extreme risk values at specific time points due to lower risks at others.

To prevent this issue, a log-sum-exp aggregation method is employed to combine time-point potentials. This method assigns proportionally greater weight to larger potential values, preventing extreme risks from being diluted during aggregation. Consequently, the presence of severe potential risk at any future time can be reliably detected. Equation (8) presents the log-sum-exp formulation used to aggregate potentials at representative points.

(8) $$R\left(a_{j}\right)=\frac{1}{\beta }log(\sum_{i=1}^{N}exp(\beta \cdot U\left(p\left(t_{k}+\tau ;a_{j}\right)\right)))$$

In this formulation, U represents the potential induced by the environment and obstacles and serves as a risk indicator. The parameter β emphasizes maximum risk to prevent large risks from being masked by averaging. R denotes the accumulated risk value obtained by weighting time-point risks along the future trajectory generated by the acceleration candidate $a_{j}$, where smaller values indicate safer trajectories.

The parameter β controls the trade-off between averaging and maximum-risk sensitivity. When β is small, the aggregation behaves similarly to an average, causing local high-risk regions to be smoothed out. As β increases, the aggregation increasingly emphasizes the largest potential values, leading to stronger avoidance of localized high-risk areas. Therefore, larger β values promote conservative behavior focused on worst-case risk, while smaller values result in smoother but potentially less responsive trajectories. In this study, β was selected to ensure sufficient sensitivity to localized risk while avoiding excessive conservativeness and instability due to noise amplification. The chosen value was determined empirically without formal optimization; thus, further tuning or optimization of β may improve performance in future work.

Nevertheless, even when acceleration variation is constrained within controllable limits, selecting minima based on marginal risk differences may lead to repeated small oscillations in speed, which is undesirable. To suppress such unnecessary oscillations, acceleration candidates within a predefined range are treated as having equivalent risk. Accordingly, candidates with nearly identical risk values are grouped into a single set, and a safe risk tolerance set is defined using Equation (9).

(9) $$R_{min}=min\;R\left(a_{j}\right),\;R_{2nd}=min\;(R\left(a_{j}\right)-R_{min})$$

Given an expansion factor m and an allowance ratio n, additional candidates are included as defined in Equation (10).

(10) $$m=n\times (R_{2nd}-R_{min})$$

The resulting risk tolerance set is defined as shown in Equation (11).

(11) $$A_{k(safe)}=\left\{a_{j}\in A_{k}|R\left(a_{j}\right)-R_{min}\leq m\right\}$$

#### 3.4.3. Cost-Function-Based Acceleration Selection

After determining the risk tolerance set, the final acceleration candidate for the next time step is selected using a cost function based on two criteria. First, after applying acceleration, the candidate $a_{j}$ is chosen such that the vehicle speed at the next time step after dt, namely, $v_{k+1}$, becomes closest to the desired speed, $v_{ref}$, as defined in Equation (12).

(12) $$v_{k+1}=v_{k}+a_{j}\tau ,\;\;V\left(a_{j}\right)=\frac{\left|v_{k+1}-v_{ref}\right|}{v_{ref}}$$

Second, the candidate $a_{j}$ is selected to minimize the difference between the previous-step acceleration $a_{k-1}$ and the current-step acceleration $a_{k}$. The acceleration variation (jerk) is evaluated using Equation (13).

(13) $$J\left(a_{j}\right)=\frac{\left|a_{j}-a_{k-1}\right|}{a_{k-1}}$$

These two criteria are combined into a unified cost function as expressed in Equation (14), where $\omega_{v}$ and, $\omega_{a}$ denote weighting coefficients that determine the contribution of each term.

(14) $$cost=\omega_{v}\frac{\left|v_{k+1}-v_{ref}\right|}{v_{ref}}+\omega_{a}\frac{\left|a_{j}-a_{k-1}\right|}{a_{k-1}}$$

Through this formulation, the system selects an acceleration candidate that reaches a speed close to the recommended target while avoiding excessive acceleration variation. Consequently, after verifying safety conditions, the system can suppress unnecessary control fluctuations and achieve stable speed control within the safe range.

It should be noted that Equations (12)–(14) involve normalization terms with respect to the reference velocity $v_{ref}$ and the previous acceleration $a_{k-1}$, which may lead to numerical instability when these values approach zero. In particular, Equation (12) may become sensitive when the reference velocity is close to zero, and Equation (13) may exhibit instability when the previous acceleration approaches zero (e.g., during constant-speed motion).

In practice, such conditions rarely dominate the planning process, as the reference velocity is typically defined based on feasible driving conditions and does not remain at zero over the planning horizon. Nevertheless, to ensure numerical stability, a small constant term is added to the denominator in implementation, preventing division by near-zero values while preserving the relative comparison among candidate trajectories.

Overall, GTS-PF-based path planning separates direction planning and acceleration selection for motion unit planning, thereby treating spatial changes for risk avoidance and temporal changes differently. Direction planning, corresponding to spatial change, determines an intuitively safe direction based solely on the gradient of the potential field at the current time step. In contrast, speed planning, corresponding to temporal change, evaluates the acceleration candidate set using multi-time-point future risk and stability cost. The cost-function constraints mitigate the abrupt speed variations observed in conventional approaches, and once risk conditions are satisfied, the method encourages driving close to the desired speed while minimizing acceleration changes. Through this procedure, the system avoids hazardous directions at the current time step while simultaneously applying prediction-based decision-making regarding future arrival positions in speed and motion unit determination. This structure enables a rapid response to sudden behavior changes in a preceding vehicle or the emergence of obstacles, while generating a stable path and maintaining a driving behavior close to the recommended speed.

Furthermore, because acceleration selection in GTS-PF follows a layer-specific future-prediction-based procedure, reducing the time update interval Δt to an extremely small value does not cause the method to converge to the same structure as path planning in a three-dimensional spatiotemporal expansion. GT-PF defines time as a continuous coordinate axis analogous to space, such that time-direction exploration is performed during spatial search, and the speed-direction gradient is inherently included in the generated spatiotemporal vector. In contrast, GTS-PF discretizes the temporal evolution into independent layer-based steps and computes a separate potential field at each time step. As a result, time does not function as an exploration variable but rather as an update index for path generation, progressing at a uniform rate, while speed planning is performed separately using an acceleration-candidate-based rule. Therefore, even as Δt approaches zero, the two methods cannot share an identical mathematical space definition.

In addition, although GTS-PF may appear similar to the receding horizon strategy of MPC in that it repeatedly performs local-scale planning, the underlying structures are fundamentally different. MPC optimizes an entire fixed-length prediction horizon to derive a control input sequence, applies only the first input, and subsequently re-optimizes the remaining horizon. In contrast, GTS-PF performs decision-making at each time step based on evaluations of potential-field gradients and risk, and completes the overall path by sequentially connecting the results from previous steps. Accordingly, GTS-PF does not repeatedly optimize the entire horizon but instead achieves predictive continuity through potential updates and candidate evaluation.

## 4. Simulation and Performance Evaluation

### 4.1. Implementation and Experimental Setup

For the quantitative evaluation of the proposed method, a scenario was designed in which the ego vehicle avoids a slow-moving preceding vehicle by performing a lane change, overtakes the vehicle, returns to the original lane, and proceeds toward the destination. Obstacle-avoidance scenarios are widely adopted in potential-field-based path planning studies, and avoidance success has been commonly used as a key criterion for evaluating path generation models. This is because the potential field framework was fundamentally developed to generate avoidance behaviors through repulsive potentials. In particular, obstacle avoidance in road environments differs from avoidance in unconstrained planar spaces, since lane-change feasibility, lateral movement into adjacent lanes, and lane-keeping behavior under non-avoidance conditions must be simultaneously considered. Accordingly, this study constructed a scenario in which the ego vehicle overtakes a slow preceding vehicle by changing lanes and subsequently merges in front of the vehicle. Although this scenario may appear to represent a simple lane-change maneuver, it actually involves a sequential combination of complex driving behaviors, including car-following behind the preceding vehicle, lane-change decision-making upon reaching a safe-distance threshold, parallel driving in the adjacent lane, and re-merging into the original lane ahead of the preceding vehicle.

**Figure 5:**
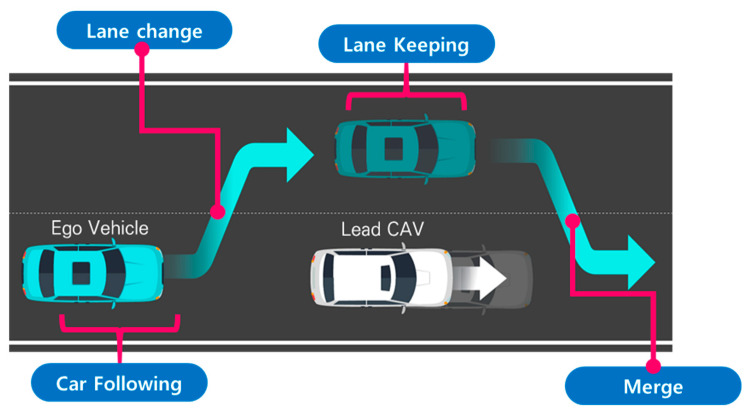
Schematic illustration of the overtaking maneuver, showing the ego vehicle’s lane-change trajectory, including the initial lane-change to the overtaking lane, acceleration to pass the leading vehicle, and the subsequent return to the original driving lane.

**Figure 6:**
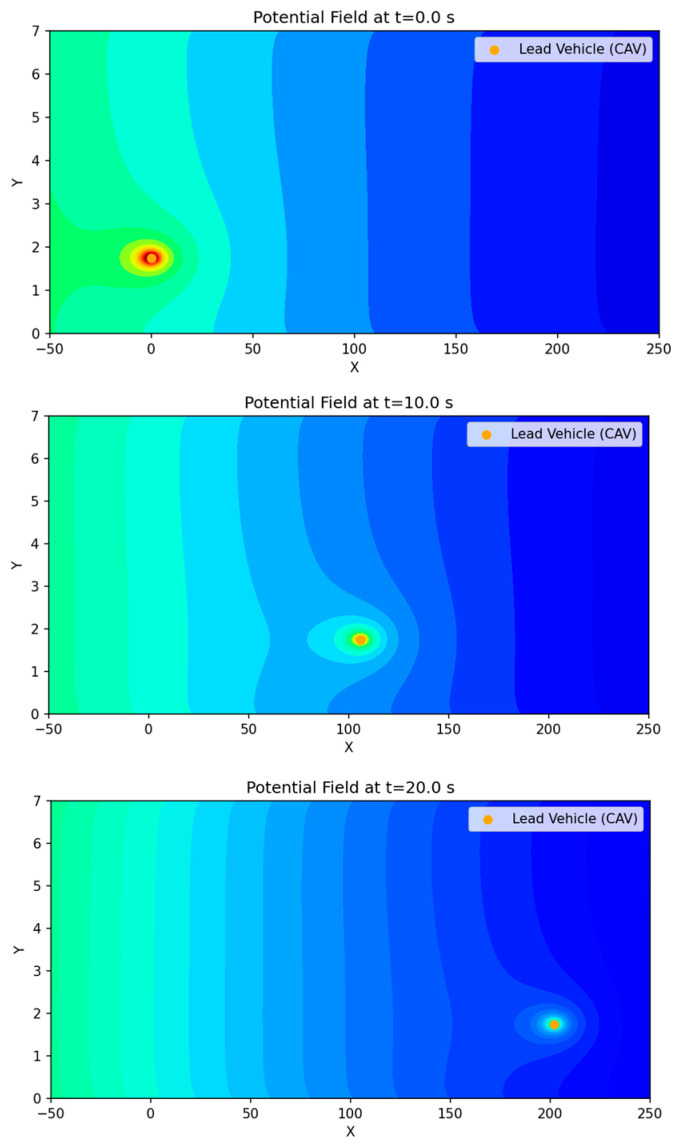
Changes in the potential field map due to leading vehicle motion. Note: The color variations are used only to visually represent changes in intensity and may differ from the actual quantitative values.

The scenario configuration and road geometry are illustrated in Figure 5. Figure 6 presents the motion of the preceding vehicle over time and the corresponding variation in the surrounding potential field map from the perspective of the ego vehicle.

As shown in the overall potential map in Figure 6, the potential distribution maintains a gradually decreasing trend toward the destination. The lane-edge regions exhibit relatively high potential values due to the boundary potential, while the lane-center region also presents a mild gradient. However, the gradient magnitude remains sufficiently small such that vehicle intrusion is not strongly restricted when influenced by other risk factors. As the preceding vehicle moves, a dynamic variation is observed in which the vehicle-induced potential region centered on the preceding vehicle shifts forward over time.

Based on this scenario, the simulation environment was configured assuming a straight two-lane one-way road segment, with a total road width of 7 m and a lane width of 3.5 m for each lane.

At the initial simulation time (t = 0), the ego vehicle is located at x = −75 m and y = 1.75 m (center of lane 1) with an initial speed of 16.66 m/s (approximately 60 km/h). The preceding vehicle is located at x = 0 m and y = 1.75 m (center of lane 1) with an initial speed of 11.11 m/s (approximately 40 km/h) and an acceleration of −0.1 m/s^2^. The objective of the ego vehicle is to safely overtake the preceding vehicle by changing to lane 2 and accelerating, and then return to the original lane to reach the destination at x = 500 m and y = 1.75 m. The road lane structure is defined as y = 0~3.5 m for lane 1 and y = 3.5~7.0 m for lane 2. Both the ego vehicle and the preceding vehicle start from the center of lane 1 (y = 1.75 m). During overtaking, the ego vehicle performs a lane change to y > 3.5 m and subsequently returns to y ≈ 1.75 m. The main parameters are summarized in Table 1.

All driving-environment information, including lane boundaries, lane-center positions, and the real-time state of the preceding vehicle, is assumed to be provided in real time by the RSU. In this study, the infrastructure-based cooperative driving framework assumes that vehicle state information is obtained through communication messages (e.g., V2I), rather than relying solely on onboard perception. Based on this information, the RSU generates reference trajectories and transmits them to individual vehicles for execution.

The proposed method focuses on decision-level path planning at the infrastructure level. Accordingly, vehicle dynamics are considered at a constraint level, including acceleration and jerk limitations, while detailed vehicle control and low-level actuation are delegated to the onboard vehicle controller.

It should be noted that communication protocols, data transmission reliability, and security mechanisms are not explicitly modeled in this study and are assumed to be handled within the underlying cooperative driving system. In addition, scalability with respect to the number of vehicles and large-scale deployment remains beyond the scope of this work and is identified as an important direction for future research.

To compare methods, evaluation metrics are required to quantitatively present the results. Table 2 summarizes the evaluation metrics adopted in this study. These metrics were selected from widely used indicators for assessing path planning performance, based on their relevance to the objectives of this work. The selected metrics can be broadly classified into safety, path quality, and computational efficiency. Safety metrics evaluate fundamental collision avoidance capability by assessing whether the collision risk was sufficiently reduced based on the distance and positional relationship with surrounding risk factors. This is also the most essential requirement for a generated path to be considered valid, since failure to avoid obstacles indicates an unsuccessful path. However, excessively conservative safety margins may lead to inefficient driving behavior, and therefore, securing an appropriate level of safety is important. Path-quality metrics were originally used in static path planning to evaluate whether the generated path is geometrically well-formed and reaches the destination without unnecessary detours. As path planning has evolved toward dynamic operation, this criterion has been extended to assess stability, dynamic feasibility, and ride comfort expected when the planned path is executed by a real vehicle.

Computation time, which represents computational efficiency, is a critical metric from the perspective of infrastructure-driven cooperative driving automation, where path computation is performed at the edge and information must be exchanged rapidly. Since computation time is sensitive to environmental variations and fluctuations in computational load, repeated measurements are required to obtain reliable quantitative values.

Among the safety metrics, the minimum TTC (time to collision) is defined as an indicator that quantifies collision risk with the preceding vehicle by jointly considering longitudinal distance and speed, expressed as the remaining time until collision if the current state is maintained. In general, a larger TTC indicates a greater safety margin. Therefore, this study compared safety performance based on the minimum TTC value, which corresponds to the most critical moment. When the longitudinal distance between two vehicles is defined as dx, the TTC can be formulated as shown in Equation (15).

(15) $$\begin{array}{c} TTC=-\frac{dx}{d^{\prime }x}\\ \left(d^{\prime }x<0\right) \end{array}$$

The interpretation of appropriate TTC threshold values varies across studies. According to a review paper that comprehensively summarized safety indicators, the desirable TTC range differs depending on the scenario considered, and threshold values reported in the literature range from 1.0 s to values above 4.0 s, as summarized in Table 3 [42]. Table 3 lists TTC thresholds reported in major prior studies, categorized by their corresponding road environments.

Accordingly, TTC is a metric that should be applied flexibly depending on road type and traffic conditions rather than relying on a single fixed threshold. In particular, Yang et al. [47] investigated TTC in lane-change situations from the perspective of ADAS (Advanced Driving Assistance System) implementation. The study reported that 95% of drivers exhibit braking responses when TTC falls below 2.8 s, and suggested 4.7 s, corresponding to the time at which 90% of drivers initiate braking, as a threshold indicating that safety can be ensured.

Meanwhile, TTC has an inherent limitation in that it can only be defined when two vehicles are traveling in the same lane and their relative longitudinal distance is decreasing. For instance, situations in which two vehicles travel at the same speed but are still considered hazardous due to insufficient separation distance cannot be adequately explained using TTC. Therefore, additional complementary metrics are required to comprehensively compare safety performance.

To address this limitation and to describe non-overlapping occupied regions even when vehicles are located in different lanes or in diagonally offset configurations, this study adopted an SDM (Safety Distance Margin) and formulated it using an expanded elliptical model based on the Minkowski sum. The most direct approach to evaluating collision and safety between vehicles is to measure inter-vehicle distance. However, distance metrics computed solely based on vehicle center points cannot sufficiently represent the actual spatial occupancy of vehicle bodies. In addition, a simple Euclidean distance metric cannot clearly distinguish the fact that vehicles traveling in parallel within lane-separated road space may have small distances yet remain relatively safe compared to diagonally positioned vehicles with similar distance values. The Minkowski sum is a technique widely used in safety analysis for determining contact in robot motion planning. Instead of explicitly computing the overlap between the occupied areas of two vehicles, it constructs an expanded safety region by combining the boundaries of both vehicles, and contact can be determined by evaluating whether the other vehicle, modeled as a point, intrudes into the expanded elliptical region [48]. This approach offers advantages such as reduced computational cost compared to rectangular intersection checking, continuity without discontinuities, and intuitive distance-based interpretation.

The SDM computation procedure is described as follows. Let dx denote the longitudinal distance and dy denote the lateral distance between two vehicles. The vehicle body dimensions are defined as $L_{ego},W_{ego}$ for the ego vehicle and $L_{cav},\;W_{cav}$ for the other vehicle. The radius of the expanded ellipse is then defined as shown in Equation (16), which combines the longitudinal and lateral radii.

(16) $$R_{x}=\frac{L_{ego}+L_{cav}}{2},R_{y}=\frac{W_{ego}+W_{cav}}{2}$$

The normalized relative distance between the two vehicles in the ellipse coordinate system is expressed as Equation (17).

(17) $$D=\sqrt{{\left(\frac{dx}{R_{x}}\right)}^{2}+{\left(\frac{dy}{R_{y}}\right)}^{2}}$$

Based on this formulation, the SDM is defined as shown in Equation (18).

(18) $$SDM=D-1=\sqrt{{\left(\frac{dx}{R_{x}}\right)}^{2}+{\left(\frac{dy}{R_{y}}\right)}^{2}}-1$$

The SDM result is a dimensionless value. When the value is 0, it indicates that the defined safety boundary for the two vehicles is just in contact, and when the value is negative, it indicates that the regions are in contact or overlapping. In this study, a vehicle width of 2 m and a vehicle length of 5 m were applied to represent vehicle body size. These values conservatively reflect typical passenger vehicle dimensions. Therefore, this indicator can be described as a safety metric that comprehensively represents diagonal distance and lateral distance.

For the maximum acceleration, which is used to represent the appropriateness of dynamic path planning in terms of path quality, Liu et al. [49], in a previous study on autonomous vehicle trajectory optimization, reported that the typical acceleration range of general vehicles is 4.0 $m/s^{2}$. They further stated that passengers experience comfort within an acceleration range of 2.0 $m/s^{2}$, while accelerations up to 6.0 $m/s^{2}$ may be achievable under emergency conditions.

Yaw rate represents the degree of change in the driving direction per unit time. Although it may appear similar to trajectory curvature in that it reflects the extent of path bending, yaw rate differs in that it is defined as a time-based rotational velocity and is influenced by both vehicle speed and trajectory curvature. Therefore, even for paths with identical curvature, inappropriate time planning may lead to variations in yaw rate and cause degradation in driving stability. Accordingly, yaw rate serves as an indicator that reflects the feasibility of executing the generated path under the planned speed profile and steering control. Let ψ denote the heading angle of the vehicle; the yaw rate $\psi^{\prime }$, defined as the time derivative of ψ, can be expressed as Equation (19).

(19) $$\psi^{\prime }\left(t\right)=\frac{d}{dt}\left\{arctan(\frac{v_{y}(t)}{v_{x}(t)})\right\}$$

Yaw rate is expressed in units of radians per unit time and represents angular velocity, indicating the rate of directional change over time. Accordingly, a large maximum yaw rate implies an instantaneous and sharp change in heading direction. A certain magnitude of yaw rate is necessary for steering when tracking a curved path; however, excessively large values suggest abrupt directional transitions. There is no universally established threshold value for yaw rate, and it is therefore employed as a supplementary indicator to assess the tendency of sudden rotational behavior.

The following section examines the variation in the GTS-PF method with respect to changes in the time update interval.

### 4.2. Time-Step Configuration and Analysis

In the GTS-PF method, the time update interval determines how rapidly the model reflects variations in the potential field environment and updates vehicle states. It also defines the fundamental motion unit used for path planning at each planning step. During a given update interval, the planned motion is assumed to remain consistent, and the risk information evaluated at the current step is assumed to be valid throughout the interval.

Although frequent field updates and rapid action changes may intuitively appear to improve safety by enabling faster responses, this is not necessarily true in practice. Excessively short update intervals may lead to continuous behavioral fluctuations and increased computational burden, which can result in unstable motion or inefficient planning. Therefore, trajectory variations were investigated by increasing and decreasing the time update interval, using 0.5 s as the baseline configuration.

#### 4.2.1. Qualitative Path Behavior Analysis of Time-Step Configuration

**Figure 7:**
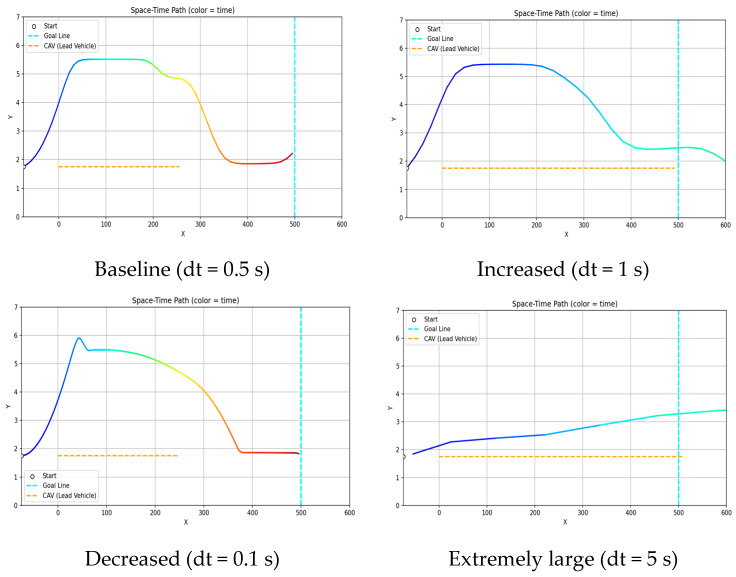
Comparison of path shapes under different time update intervals. Note: The gradient line represents the temporal progression along the trajectory, and color variations are used for visual distinction only.

Under the baseline update interval (dt = 0.5 s), a relatively stable trajectory was obtained, enabling smooth lane-center alignment and appropriate timing of lane return and merging. When the update interval was increased (dt = 1.0 s), the trajectory became smoother; however, merging into the original lane tended to be delayed, and fine adjustments for precise lane-center alignment were limited. Conversely, when the update interval was reduced (dt = 0.1 s), lane changes were executed accurately, but the shortened update interval caused small control adjustments to accumulate, resulting in oscillation-like instability in certain segments. Although dynamic constraints are defined in relation to the update interval, an excessively short decision cycle can cause repetitive minor corrections within the constraint range, leading to trajectory instability. In extreme cases where the update interval was excessively large, delayed lane-change execution led to collision risks or difficulty maintaining lane-center alignment. Based on these qualitative observations, the trajectory generated at dt = 0.5 s was determined to be the most appropriate. Figure 7 illustrates the trajectory characteristics under different update intervals, supporting these observations.

#### 4.2.2. Quantitative Performance Metrics Analysis of Time-Step Configuration

To quantitatively analyze the effects of varying the time update interval in GTS-PF, performance metrics corresponding to update intervals varied in 0.1 s increments are summarized in Table 4.

**Figure 8:**
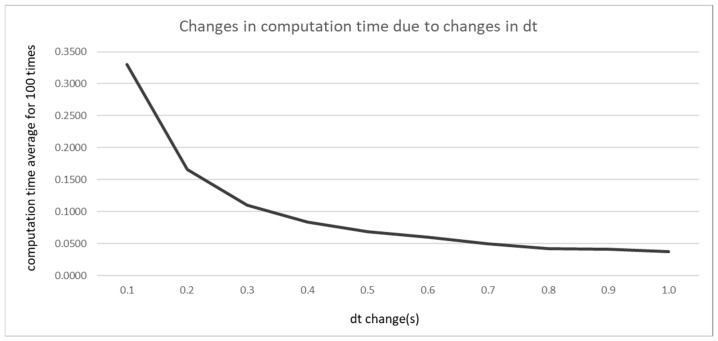
Changes in computation time with respect to the time update interval.

Average computation time was measured by varying only dt under identical conditions and conducting 100 repeated trials to account for variations in PC computational load. As the update interval increased, the number of intermediate planning steps required to reach the destination decreased, leading to a reduction in computation time. However, this reduction does not continue indefinitely; beyond a certain threshold, the rate of decrease diminishes and converges toward a steady level. Figure 8 illustrates computation time as a function of dt.

As the update interval increased, GTS-PF exhibited a tendency to maintain larger conservative distances from obstacles. However, excessively large intervals delayed avoidance initiation, resulting in inefficient merging behavior. Conversely, reducing the update interval enabled avoidance closer to the obstacle but generated abrupt accelerations and reduced safety margins. TTC generally increased with larger update intervals, whereas SDM did not show a monotonic trend. This difference arises from the nature of the metrics: TTC reflects longitudinal time to collision within the same lane and increases when risk is detected earlier and lane changes are initiated sooner. In contrast, the SDM evaluates spatial contact at the closest relative configuration, typically occurring during diagonal alignment or while overtaking in the adjacent lane.

Speed variations are regulated through the acceleration candidate set, while rotational motion is constrained by a yaw-rate limit proportional to the update interval. When the update interval is small, increased sensitivity to risk and frequent directional adjustments lead to unstable behavior in certain segments, resulting in higher maximum composite acceleration despite remaining within yaw-rate constraints.

Overall, considering trajectory stability, lane-center maintenance, composite acceleration levels, and computation-time behavior, dt = 0.5 s was identified as the most appropriate baseline in the present scenario. Therefore, subsequent comparisons between GTS-PF and GT-PF adopt dt = 0.5 s as the representative update interval.

### 4.3. Path Behavior Analysis Under Baseline Conditions

#### 4.3.1. Qualitative Path Behavior Analysis of Path Behavior Under Baseline Conditions

To evaluate the performance of the proposed GTS-PF method, the previously developed GT-PF-based path planning approach was adopted as a benchmark. Under identical road configurations and initial conditions, the trajectory outputs of both methods were compared to examine safety performance, path quality, and computational characteristics resulting from different treatments of temporal information. Because the two approaches model time differently, their trajectory evolution and avoidance strategies differ even under the same scenario. The trajectory shapes generated by GT-PF and GTS-PF were therefore directly compared to analyze how structural differences between the two methods manifest in actual path behavior.

In addition to the GT-PF benchmark, an additional comparison was conducted using the RRT -STRF trajectory generation approach to provide a broader context for evaluating the proposed method. The RRT -STRF method explores the spatiotemporal space through randomized sampling and evaluates candidate trajectories using a safety-oriented risk field. Unlike the gradient-based exploration adopted in GTS-PF, the RRT -STRF approach relies on stochastic sampling to construct feasible trajectories while considering vehicle dynamics and environmental constraints. The RRT -STRF baseline was implemented under the same simulation conditions, enabling a consistent comparison of trajectory behavior and computational characteristics across the evaluated methods.

**Figure 9:**
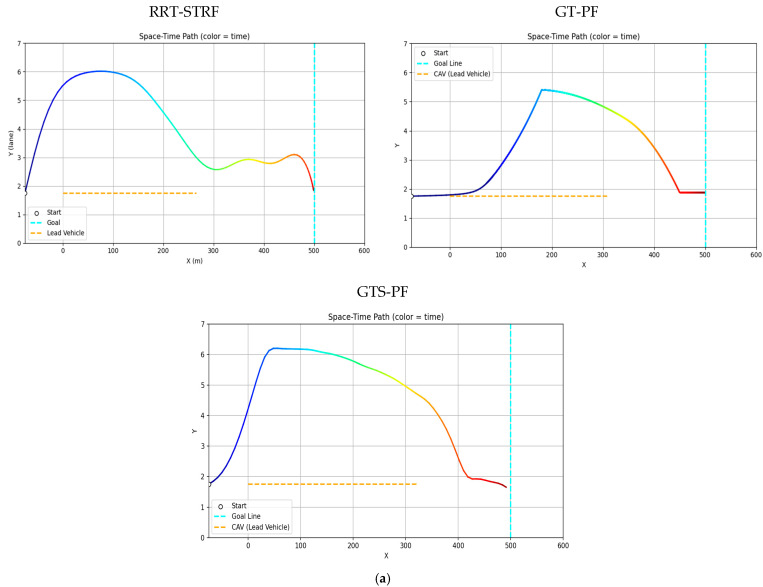

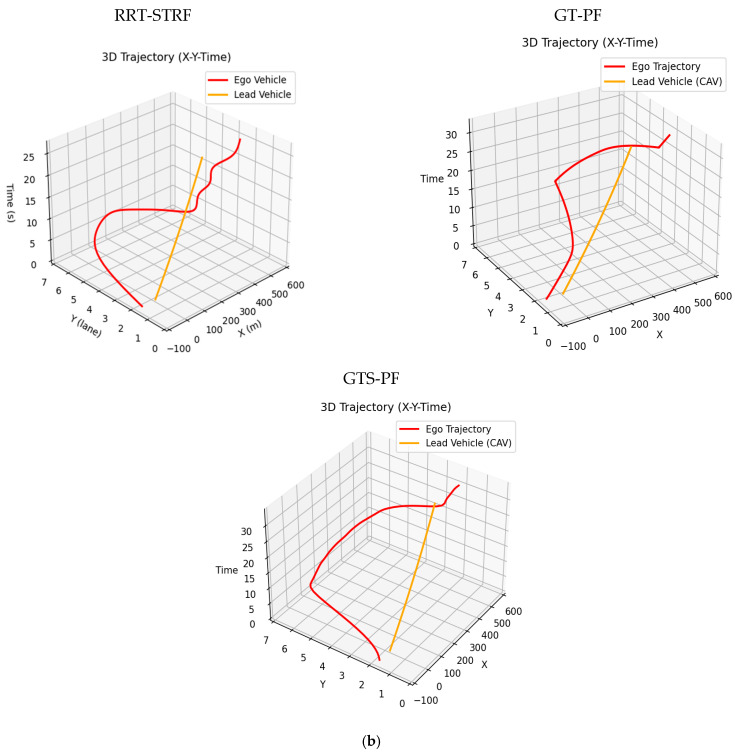

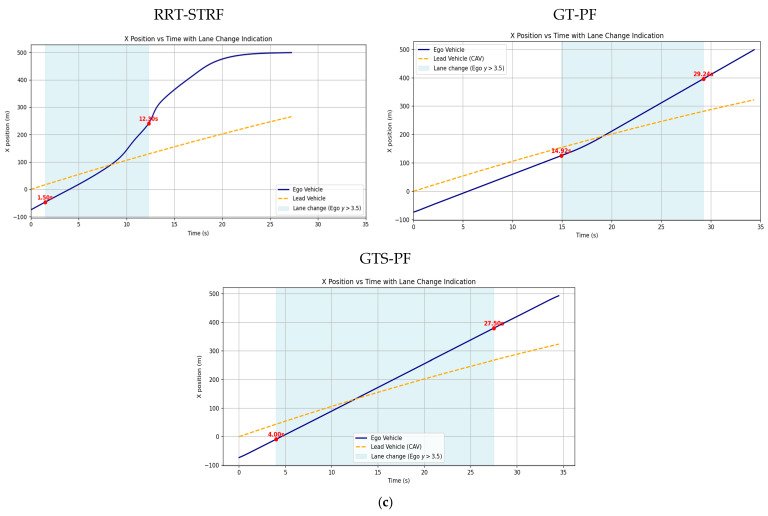
Spatiotemporal trajectories of ego and lead vehicles: (**a**) top-down view of the vehicle trajectories in the spatial domain (x-y plane); Note: The gradient line represents the temporal progression along the trajectory, and color variations are used for visual distinction only. (**b**) spatiotemporal paths represented in a three-dimensional space (x-y-t); (**c**) time–distance plot (t-x).

**Figure 10:**
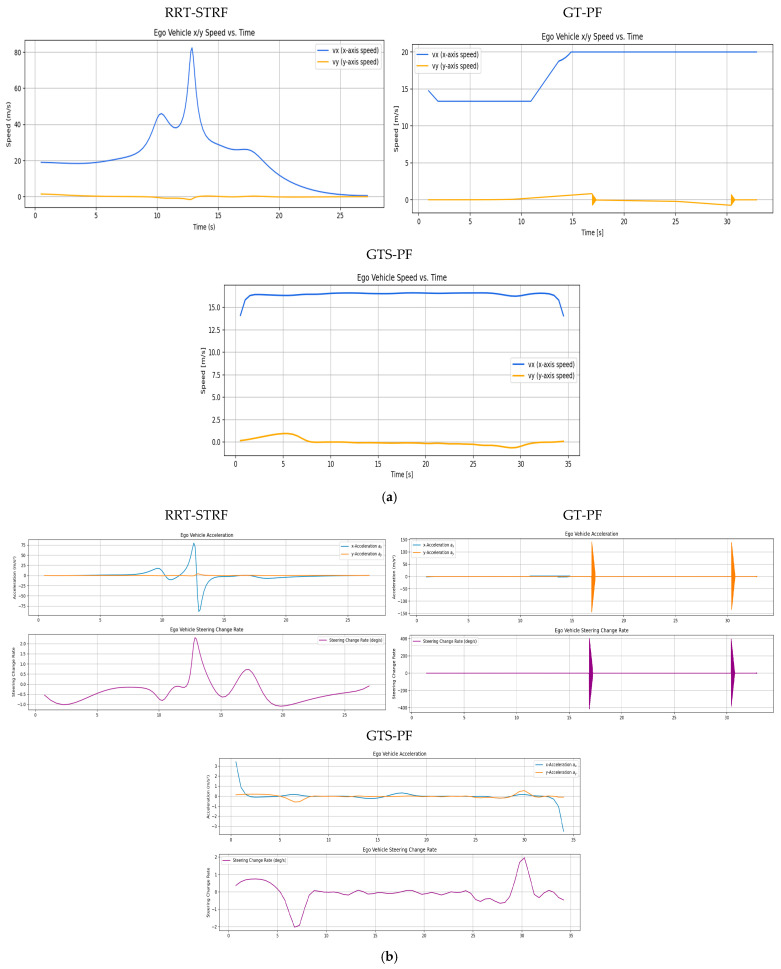
Velocity, acceleration, and steering variation by experiment: (**a**) longitudinal and lateral velocity profiles; (**b**) acceleration and steering angle variation.

Figure 9 and Figure 10 illustrate the avoidance trajectories produced by the three methods. In all cases, the ego vehicle performs a lane change to overtake the preceding vehicle, maintains longitudinal separation, and subsequently returns to the original lane before reaching the destination. However, distinct differences arise due to the underlying planning mechanisms. The RRT-STRF method explores the spatiotemporal space through randomized sampling, which can result in locally irregular trajectory structures or abrupt transitions depending on the sampling density. The GT-PF method maintains the initial lane before initiating a lane change based on predicted future vehicle states, whereas RRT-STRF and GTS-PF initiate the lane-change maneuver earlier by prioritizing current-state safety assessment.

In Figure 9c, the blue segment indicates the duration spent in the adjacent lane. RRT-STRF performs a lane change at approximately 1.5 s and returns to the original lane at around 12.3 s. GT-PF follows the preceding vehicle for a period, initiates a lane change at approximately 14 s, completes overtaking around 29 s, and then returns to the original lane. In contrast, GTS-PF initiates the lane change earlier, around 4 s, remains in the adjacent lane until overtaking is completed, and returns to the original lane at approximately 27 s in terms of longitudinal position. The earlier lane-change initiation in GTS-PF is attributed to the uncertainty in long-term predictions; the method prioritizes conservative risk avoidance based on current-state safety assessment. Whereas GT-PF utilizes predicted future vehicle positions to identify and enter anticipated safe gaps, GTS-PF selects directions deemed safe at the current planning step. The longer duration spent in the adjacent lane reflects a conservative return strategy, in which re-entry occurs only after sufficient overtaking margin is established. It should be noted that the duration spent in the adjacent lane does not directly correspond to a risk exposure metric in this study. Unlike interpretations that treat the adjacent lane as an inherently hazardous region, the proposed framework evaluates safety based on quantitative criteria such as TTC and SDM, rather than dwell time alone. In this context, a shorter adjacent lane occupancy does not necessarily imply higher safety.

The difference in adjacent lane dwell time between RRT-STRF, GT-PF and GTS-PF reflects distinct overtaking strategies. RRT-STRF and GT-PF tend to minimize adjacent lane occupancy by relying on predicted future gaps and executing relatively aggressive entry and return maneuvers. In contrast, GTS-PF initiates the lane change earlier and maintains a stable trajectory in the adjacent lane until sufficient longitudinal clearance is achieved. This results in longer adjacent lane occupancy but provides an increased temporal margin and reduces dependence on long-term prediction accuracy. Therefore, the longer adjacent lane duration observed in GTS-PF represents a conservative and risk-aware strategy rather than a decrease in safety.

Figure 10 presents longitudinal and lateral velocity, acceleration, and steering profiles. Compared to RRT -STRF, which may exhibit irregular variations due to its sampling-based trajectory construction, both GT-PF and GTS-PF produce more structured motion profiles. GT-PF maintains reduced speed while following the preceding vehicle and subsequently accelerates sharply after overtaking to clear the risk region. In contrast, GTS-PF exhibits smaller overall speed variation, particularly maintaining relatively low speed during the 9–28 s interval, where the potential gradient is mild. After returning to the original lane at approximately 26 s, speed increases slightly in response to changes in the potential field influenced by a following vehicle. In terms of steering behavior, GTS-PF produces more frequent minor steering adjustments, whereas GT-PF demonstrates more abrupt steering transitions associated with movement toward predicted future safe zones.

GT-PF incorporates predicted future vehicle positions within a continuous spatiotemporal framework, allowing entry into regions that are currently risky if they are predicted to become safe, or avoidance of regions predicted to become hazardous. In contrast, GTS-PF bases directional decisions on current-layer safety conditions, resulting in a stronger tendency toward immediate avoidance maneuvers when encountering risk. Compared to both RRT -STRF and GT-PF, this approach leads to smoother and more consistent trajectory evolution without relying on stochastic sampling or post-processing.

#### 4.3.2. Quantitative Performance Metrics Analysis of Path Behavior Under Baseline Conditions

The safety evaluation in Table 5 assesses collision occurrence by examining physical contact or overlap using the SDM and verifies the longitudinal safety margin using the minimum TTC. The TTC threshold follows the lane-change safety criterion suggested by [47], requiring TTC to remain above 4.7 s. SDM values below 0.0 indicate collision, SDM = 0.0 corresponds to surface contact, and SDM values greater than 0.0 indicate sufficient separation. During the scenario, the vehicles occasionally form diagonal relative configurations or travel side by side in adjacent lanes, resulting in locally reduced inter-vehicle spacing. Across all methods, including RRT-STRF, GT-PF, and GTS-PF, the evaluated safety indicators satisfy the predefined thresholds, indicating that all generated trajectories remain within acceptable safety limits, and no violation of the safety criteria is observed.

Table 6 presents the path-quality comparison between the two methods. The threshold for maximum acceleration was set to 4.0 m/s^2^ based on the typical acceleration range reported by [4]. Although no explicit upper bound was imposed on yaw rate, it was included as an auxiliary metric to examine steering-related driving characteristics. Unlike GT-PF, which discretely updates velocity along each unit vector, GTS-PF performs acceleration-based motion planning, enabling smoother and more continuous velocity profiles. Consequently, abrupt velocity transition points observed in RRT-STRF and GT-PF were mitigated, and the maximum acceleration remained within the feasible range for conventional vehicles.

The maximum yaw rate, reported for reference, was also reduced under GTS-PF compared to GT-PF. This result reflects the dynamic driving behavior of the proposed approach. GTS-PF suppresses abrupt changes and yields a more stable driving pattern with reduced fluctuations in both speed and steering. Avoidance and lane-return maneuvers are connected through smooth, continuous curves rather than sudden steering actions, and the vehicle maintains nearly constant speed unless additional risk factors emerge. This behavior arises from the acceleration-selection strategy, in which candidate accelerations are evaluated based on future risk at the expected arrival position, and among candidates with similar risk levels, those requiring smaller steering variation and achieving speeds closer to the desired reference velocity are prioritized. As a result, unnecessary speed oscillations are reduced except in safety-critical segments. Since the direction is determined first based on instantaneous safety, and speed control acts as a supportive mechanism for risk avoidance, an initially safe directional choice naturally leads to reduced risk in subsequent predicted arrival positions, allowing stable speed maintenance.

For the computation-time results in Table 7, average values obtained from 100 repeated runs under identical conditions were used to reduce sensitivity to variations in PC workload. The experiments were conducted on an Intel i7-12700K with 16 GB RAM, considering a single preceding vehicle moving linearly and the corresponding ego-vehicle trajectory. In practical cooperative driving systems, path planning at edge RSU devices must operate under more constrained computational resources while handling multiple vehicles and dynamic traffic environments. For the computation-time evaluation, all algorithms were executed under identical simulation conditions, and the reported runtime corresponds to the average processing time required to generate a reference trajectory for the overtaking scenario. Therefore, reducing computation time is critical for real-time applicability.

Based on the 100-run average, GTS-PF achieved approximately 80.98% reduction in computation time compared to GT-PF and 99.17% reduction compared to RRT-STRF. Although the computation time of GTS-PF is influenced by the number of layers determined by the time update interval Δt, this comparison adopted dt = 0.5 s, which exhibited the most stable performance in prior analysis. GT-PF does not operate with a fixed time step and instead expands unit time vectors with variable increments. Thus, even when planning over the same prediction horizon, GTS-PF maintains predictable computation time proportional to the number of layers, whereas GT-PF may exhibit variability due to fluctuations in its update increments.

It should be noted that the reduction in computation time is not achieved through a trade-off with safety. In this study, safety is enforced as a constraint, where all candidate trajectories are required to satisfy predefined TTC and SDM thresholds prior to selection. The final trajectory is then chosen among the feasible set of safe candidates based on efficiency criteria. Therefore, the observed reduction in computation time reflects improved efficiency within the set of safety-compliant trajectories, rather than a relaxation of safety requirements.

Overall, GTS-PF not only preserves the lightweight nature of potential-field-based planning but also reduces computational burden compared to GT-PF and RRT-STRF. In addition, safety and dynamic feasibility metrics remained within acceptable ranges, and GTS-PF demonstrated a more conservative driving tendency, initiating avoidance earlier, maintaining larger clearance margins, and providing longer response time. Furthermore, since all trajectories satisfy predefined safety thresholds, the reduction in computation time enables faster responses to dynamic traffic conditions and allows the system to incorporate additional environmental factors within the same time budget, thereby enhancing practical applicability in real-time cooperative driving scenarios.

### 4.4. Sensitivity and Extension Analysis

To evaluate the generalizability of the proposed path planning approach, additional simulations were conducted under various modified initial conditions beyond the main driving scenario. Through these extended experiments, we aimed to qualitatively verify the applicability and safety performance of GTS-PF under diverse driving environments. Table 8 summarizes the extended simulation scenarios considered for this sensitivity analysis.

#### Performance Under Diverse Traffic Conditions

**Figure 11:**
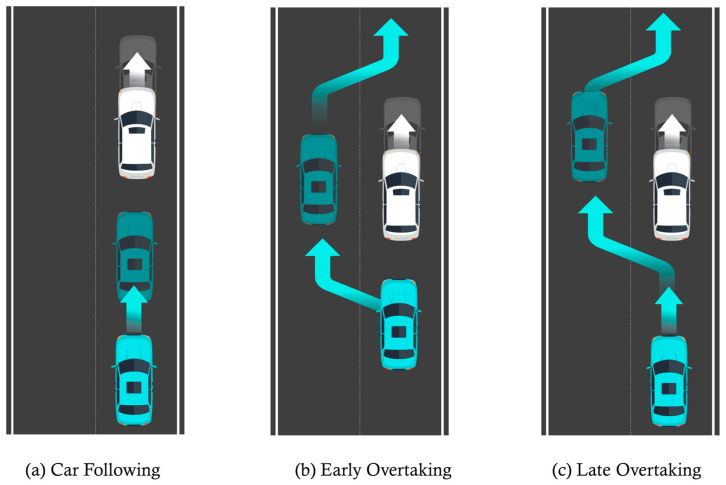
Additional sub-scenarios for evaluating the proposed method: (**a**) car-following; (**b**) early overtaking; and (**c**) late overtaking.

**Figure 12:**
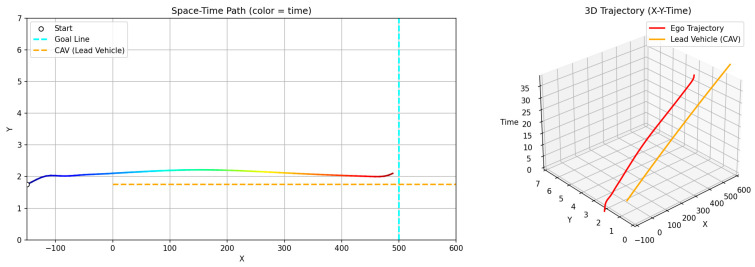
Scenario A: Following without overtaking (both 60 km/h).

**Figure 13:**
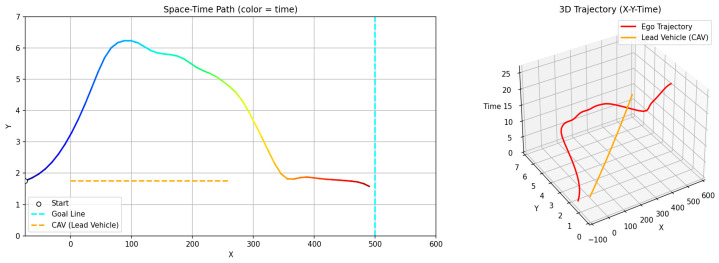
Scenario B: Early overtaking (ego: 80 km/h, lead: 40 km/h).

**Figure 14:**
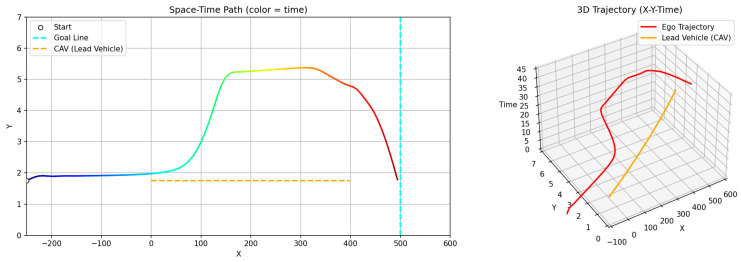
Scenario C: Delayed overtaking (longer initial gap: 150 m).

Figure 11 illustrates the scenarios constructed for the two-vehicle configuration. For scenarios (a), (b), and (c) in Figure 11, Figure 12, Figure 13 and Figure 14 present the planar trajectories generated by GTS-PF, as well as their spatiotemporal trajectory representations.

First, a scenario was assumed where both the preceding vehicle and the ego vehicle start at the same speed (60 km/h), and the preceding vehicle maintains a constant cruising speed throughout the road segment. In this case, as shown in Figure 11a, the ego vehicle follows the preceding vehicle while maintaining a safe distance, without initiating overtaking, and remains aligned with the lane center. Figure 12 presents the corresponding simulation results, demonstrating that this driving behavior is maintained continuously without lane departure until the ego vehicle reaches the destination after the preceding vehicle exits the simulation domain. This scenario indicates that the proposed GTS-PF framework can implicitly determine whether overtaking is necessary. When the preceding vehicle maintains a sufficiently high speed such that safe and efficient following is possible without excessive deceleration or inefficient motion, the ego vehicle avoids unnecessary lane changes and continues stable lane-following while maintaining appropriate spacing and speed. Therefore, under low-risk conditions, GTS-PF is able to generate a stable lane-keeping trajectory.

Figure 11b, together with the corresponding simulation result in Figure 13, represents a case where the ego vehicle begins driving at a higher speed (80 km/h) than the preceding vehicle (40 km/h). In this condition, a collision may occur if neither rapid deceleration nor a lane-change maneuver is performed. Accordingly, the ego vehicle does not remain in a following state but initiates an immediate lane-change maneuver in response to the increased collision risk.

Conversely, Figure 11c and its simulation result in Figure 14 represent a case with a significantly larger initial inter-vehicle distance, where the ego vehicle starts 150 m behind the preceding vehicle. In this scenario, the ego vehicle continues lane-keeping for a longer duration and initiates a lane-change maneuver only after entering the high-risk region influenced by the preceding vehicle. To further quantify these behaviors, the safety indicators for the sub-scenarios are summarized in Table 9.

In scenario Figure 11a, TTC is not defined because no closing conflict occurs when the ego vehicle maintains the recommended speed, while the SDM remains consistently large (28.286), confirming stable car-following behavior. In scenario Figure 11b, the combination of a short initial distance and high relative speed leads to a reduction in TTC to 4.784 s, approaching a near-critical condition; however, the rapid lane-change maneuver prevents further reduction in the safety margin, with the SDM remaining positive (1.24). In scenario Figure 11c, the lane change is initiated before a significant decrease in TTC, resulting in a relatively large TTC value (21.98 s) and maintaining a positive SDM (1.02). These results indicate that the proposed method adapts its behavior depending on traffic conditions, either maintaining stable lane-following or initiating timely lane-change maneuvers while preserving non-contact safety margins.

These results indicate that GTS-PF determines appropriate decision timing and behavioral policy for lane-change and overtaking maneuvers. The ego vehicle does not attempt overtaking solely because the preceding vehicle travels at a lower speed; instead, overtaking is executed only when the inter-vehicle gap becomes sufficiently small such that maintaining a desired cruising speed or safe distance is no longer feasible. Collectively, these scenarios demonstrate that GTS-PF can generate situation-dependent trajectories, including stable lane-center following as well as proactive lane-change and overtaking when required. This suggests that the proposed method can be applied across various speed and spacing conditions, supporting a broad range of driving behaviors, such as safe lane-keeping, following, and overtaking. Therefore, the proposed approach exhibits responsiveness and adaptability to diverse and general real-world traffic conditions.

**Figure 15:**
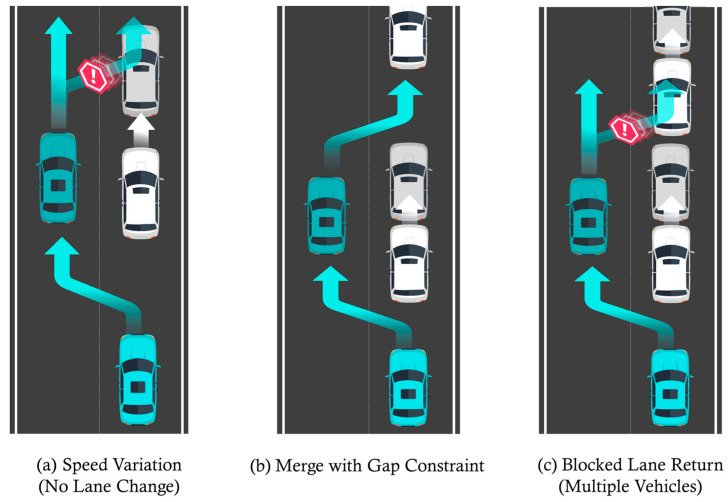
Additional sub-scenarios for evaluating the proposed method: (**a**) speed variation with no lane return; (**b**) merge with gap constraint; and (**c**) blocked lane return involving multiple vehicles.

**Figure 16:**
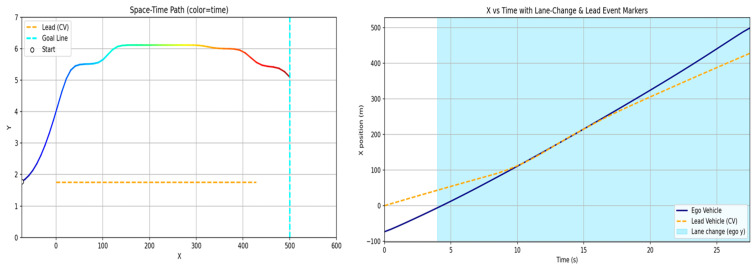
Sub-scenario A: Speed variation (no lane return).

**Figure 17:**
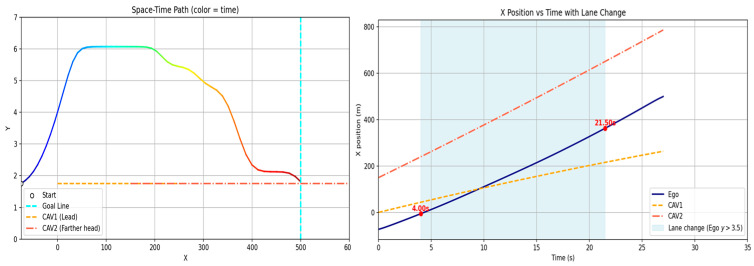
Sub-scenario B: Merge with gap constraint.

**Figure 18:**
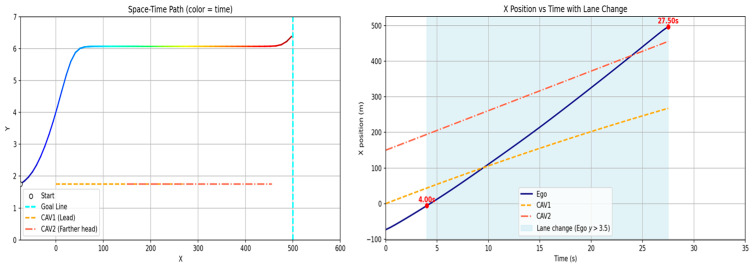
Sub-scenario C: Blocked lane return (multiple vehicles).

While the scenarios in Figure 11 modify only the speed and spacing conditions within the same environment, the scenarios in Figure 15 further extend the evaluation by varying both the behavior of surrounding vehicles and the number of vehicles present. Figure 16, Figure 17 and Figure 18 show the trajectories generated by applying GTS-PF to scenarios (a), (b), and (c) in Figure 15.

Figure 15a represents a case where unexpected behavior of the preceding vehicle makes returning to the original lane after overtaking unsafe, thereby requiring the ego vehicle to continue driving in the adjacent lane. Figure 16 shows the corresponding simulation trajectory, where the ego vehicle attempts to return after overtaking, but a sudden change in the preceding vehicle’s speed prevents sufficient merging clearance, resulting in delayed lane return. In such a case, aggressively accelerating to merge ahead may introduce significant risk; therefore, the ego vehicle maintains stable driving in the overtaking lane for an additional segment rather than forcing an immediate return. In real traffic environments, surrounding vehicles rarely maintain constant speed or constant acceleration and may exhibit diverse speed variations. Such behavior is difficult to address using static path planning, whereas dynamic path planning enables safe adaptation by recognizing and responding to these changes.

Figure 15b represents a merging scenario, where the ego vehicle attempts to merge between two vehicles and must adjust its spacing with both the leading and following vehicles in order to return to the original lane. Scenario (c) is similar, but at the time of the lane-return decision, the gap between the two vehicles occupying the target lane is insufficient, causing lane return to be delayed or infeasible. Figure 17 presents the simulation results for Figure 15b, assuming that two vehicles exist ahead, the foremost vehicle travels at a sufficiently high speed, and the spacing between the two vehicles is large enough to allow merging. To safely merge between the two vehicles, the ego vehicle must maintain adequate clearance both in front and behind, thereby requiring simultaneous gap management with multiple vehicles rather than a single target. The simulation results show that the ego vehicle successfully merged into the gap and maintained appropriate longitudinal distances with both vehicles while continuing stable driving.

Figure 18 presents the simulation result for Figure 15c, where the gap between the two vehicles is too narrow to provide a sufficient safety margin for merging. In this case, the ego vehicle did not execute a lane-change maneuver that would violate safe-distance constraints and instead continued driving in the original lane. This result indicates that the proposed method can reject lane-change decisions when safe longitudinal clearance cannot be ensured. To quantitatively assess these behaviors, the safety indicators for the extended scenarios are summarized in Table 10.

In scenario Figure 15a, the ego vehicle maintains an adequate TTC (7.56 s before lane-change decision) but refrains from lane return as the SDM is expected to decrease excessively, indicating a conservative decision to preserve safety margins under dynamic conditions.

In scenario Figure 15b, the ego vehicle successfully executes a merging maneuver between two vehicles; however, the TTC with respect to the rear vehicle in the target lane decreases to approximately 0.96 s, representing a near-critical interaction. At the same time, sufficient longitudinal clearance is maintained with the front vehicle, resulting in asymmetric safety conditions during the maneuver. Despite the reduced TTC, the SDM remains positive for both front and rear interactions, indicating that no physical overlap occurs.

In scenario Figure 15c, the lane-change maneuver is not executed due to the anticipated reduction in SDM caused by the insufficient gap between the two vehicles in the target lane. This demonstrates that the proposed method can reject infeasible merging decisions when adequate safety margins cannot be ensured. Overall, the results confirm that the proposed framework adapts its decision-making according to both current safety conditions and multi-vehicle interactions, prioritizing conservative and feasible trajectory selection.

Although the proposed approach was initially designed and implemented based on a single baseline scenario, the above extended experiments confirm its stability and adaptability under diverse traffic conditions. The results demonstrate that the algorithm can effectively adjust trajectories in response to dynamic factors, such as preceding vehicle speed changes, multi-vehicle interactions, and merging feasibility. In particular, the proposed method shows the capability to flexibly adapt trajectory patterns and determine appropriate lane-change timing and lane-return decisions depending on situational demands. However, this study is limited to straight road segments, and further research is required to extend the applicability to more complex environments, including intersections, curved road geometries, and variable lane configurations.

### 4.5. Robustness of Path Planning Under Sensing Noise and Communication Uncertainty

**Figure 19:**
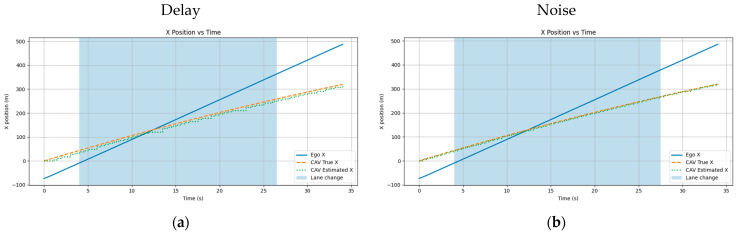
The x–t plots of actual and perturbed leading vehicle trajectories and the corresponding ego response under sensing and communication uncertainty: (**a**) communication delay and packet loss scenario; (**b**) sensing noise scenario.

To evaluate robustness under sensing and communication uncertainty, additional experiments were conducted by incorporating both communication delay/loss effects and sensing noise into the vehicle state information. The corresponding trajectory results are illustrated in Figure 19. Despite degraded conditions, the overall trajectory structure is preserved, and no abrupt instability or infeasible behavior is observed.

Specifically, two types of uncertainty scenarios were considered under the unified context of the impact of sensing and communication uncertainty on path planning. Figure 19a represents the case with communication delay and packet loss, where state information is subject to an average delay of approximately 0.3 s, with additional temporal variation (jitter) on the order of 0.08 s. Packet loss occurs with a probability of approximately 10%, and in such cases, the most recently received state is retained without applying predictive delay compensation. This configuration reflects a conservative communication condition in which delayed and intermittent updates must be handled without explicit prediction.

Figure 19b represents the case with sensing uncertainty, where the vehicle state is affected by both measurement noise and low-frequency bias. The position measurement includes longitudinal noise on the order of 0.8 m and lateral noise of approximately 0.05 m, while velocity measurements are perturbed by noise of approximately 0.5 m/s. In addition, slowly varying bias components are introduced to both position and velocity to emulate realistic sensor drift over time.

These configurations provide a controlled evaluation of the proposed method under realistic uncertainty conditions, encompassing both communication-induced degradation and sensing imperfections within a unified experimental framework.

The corresponding safety indicators are summarized in Table 11. Compared to the nominal condition, the minimum TTC remains unchanged across the considered uncertainty scenarios, indicating that longitudinal collision risk is effectively managed even under degraded sensing and communication conditions.

In contrast, a slight reduction in SDM is observed, particularly during close-proximity interactions when the ego vehicle passes near surrounding vehicles. This reduction can be attributed to inaccuracies in position estimation caused by sensing noise and communication delay, which may lead to temporarily reduced inter-vehicle spacing in certain segments. While the overall trajectory remains feasible and stable, these results suggest that lateral or near-field interactions are more sensitive to uncertainty than longitudinal spacing control.

These findings indicate that, although the proposed method maintains stable longitudinal safety margins, additional caution may be required in close-range interactions, where small estimation errors can affect relative spacing.

It should also be noted that identifying acceptable uncertainty bounds and quantitatively defining safety margins under varying noise and delay conditions represent meaningful research directions. However, such analysis is beyond the scope of the present study.

Furthermore, this experiment was conducted without predictive delay compensation and assumes a conservative communication setting with intermittent updates. The results therefore demonstrate that the GTS-PF framework can maintain stable behavior under moderate sensing and communication uncertainty.

However, the current framework does not explicitly perform re-planning under asynchronous or delayed inputs. Instead, trajectory generation is updated sequentially based on the most recently available information. As a result, handling fully asynchronous data streams or designing dedicated re-planning strategies under severe communication degradation remains an important topic for future work.

## 5. Conclusions

### 5.1. Discussion of Results

Motivated by the need for lightweight path planning to support inter-vehicle negotiation in cooperative driving automation and by the ongoing development of potential-field-based path planning techniques, numerous studies have investigated potential-field-based approaches that overcome limitations of conventional methods and enable dynamic path planning. Among these, the GT-PF-based planning method proposed in prior work achieved dynamic path planning with relatively low computational resource consumption by conducting gradient-based planning in the spatiotemporal domain. However, because the planning procedure was based on constant-velocity motion, the resulting acceleration profiles were expressed in an unrealistic manner that differs from actual vehicle control. Moreover, under settings where the time movement unit is not fixed, frequent behavior updates at very small update intervals may accumulate, producing abrupt variations that exceed the maximum acceleration threshold. To address these issues, we propose the GTS-PF method and implement a dynamic planning strategy that, within a sequentially updated time-layer environment, determines the motion unit by evaluating the safety of future reachable positions and the cost induced by dynamic changes over a reasonable set of acceleration candidates, and executes the selected motion through continuous speed variation.

Starting from the basic interaction between two vehicles, we conducted simulations of multi-vehicle scenarios, and we derived qualitative trajectory shapes to confirm the applicability of the proposed method. In addition, we varied the update time step and observed corresponding changes in trajectory behavior, and by comparing the proposed model against the prior method under the same scenario, we confirmed that the proposed approach maintains lightweight characteristics and safety while improving path quality to a level that is feasible for practical implementation.

The comparison results indicate that computational time decreases relative to the existing method, thereby preserving lightweight operation, and that the fixed update time step enables consistent and predictable computation time. In terms of safety, indicators such as SDM and TTC satisfy the specified threshold ranges. However, these safety indicators increase compared with those obtained by GT-PF, revealing conservative behavior characterized by earlier avoidance initiation, larger distance margins, and longer maintained response time. This tendency arises because GT-PF can exploit unoccupied regions predicted at future times and thus approach closer to risky space, whereas GTS-PF prioritizes the occupancy state of the current layer and avoids regions with uncertainty at an earlier stage.

The path-quality issue associated with maximum acceleration exceeding the threshold, which was problematic in the existing method, was improved to a practically implementable level. Whereas the previous approach planned discrete speeds at each unit vector and produced excessive acceleration at transition points—thereby deviating from realistic vehicle speed planning—GTS-PF realizes continuous speed variation through acceleration-based planning and exhibits behavior that is closer to actual vehicle speed changes. The supplementary yaw-rate measure also decreases, indicating a more stable path with fewer abrupt heading changes.

Furthermore, because GTS-PF applies potentials at specific update instants on a time-layer basis, the redundant incorporation of future information observed in GT-PF—stemming from vehicle formulations that already embed future-state effects—can be reduced. Conversely, elements that are difficult to represent solely by embedding future changes into a potential formulation, such as occupancy-state transitions, are directly reflected via layer updates, enabling natural handling of environmental changes. Overall, GTS-PF maintains a short computation time while alleviating the infeasible acceleration fluctuations seen in GT-PF, and it provides continuous speed planning with higher practical followability. These results suggest that the time-layer structure and acceleration-based control improve field suitability of the planning method.

In conclusion, the method developed in this study achieves reduced computation time as well as the safety and dynamic path quality required for driving. This research process does not merely refine a subset of the path-search pipeline, but begins by redefining and managing the force formulation exerted by vehicles within the field. Accordingly, it is meaningful in that it addresses the overall procedure and the systematic refinement of limitations, including the planning method that searches in the field constructed by the proposed formulation. In addition, strategies designed to mitigate the dynamic instability observed in GT-PF are incorporated into GTS-PF, resulting in a path generation algorithm capable of planning paths and controlling speed under realistic constraints. In summary, this study contributes by demonstrating the applicability of cooperative path generation through lightweight vehicle potential formulation, safety-aware extension to dynamic planning for moving agents, and improved dynamic path quality achieved via time-sequential planning and acceleration-based control.

### 5.2. Evaluation from the Perspective of Infrastructure Application

Based on the results of this study, we discuss the feasibility of applying the proposed method as a reference-path planning approach for cooperative driving automation in an infrastructure-based RSU environment. First, regarding information availability for constructing the potential field, the proposed field can be generated using realistic inputs under the assumption that future vehicle behaviors are shared through cooperation and remain valid within the prediction horizon. In addition, since road geometry and lane-related information are inherently available to the infrastructure, the interaction distribution over the entire road region can be represented in a straightforward manner. From a computational perspective, the proposed method does not optimize the entire horizon; instead, it performs repeated local decision-making based on time-layer segmentation, which makes it suitable for RSU-level implementation under limited computational resources. Furthermore, the generated path is intended as a reference trajectory for negotiation purposes and is explicitly separated from the vehicle control layer. The transmitted data provides a description of driving behavior, while actual vehicle control is executed onboard. Accordingly, the proposed method does not require vehicle-specific control characteristics or internal controller structures to be incorporated into either computation or transmitted data, as such considerations are handled by each vehicle. This separation enables lightweight operation in terms of both data transmission size and computational resource requirements. Even when the number of vehicles increases or complex road alignments are introduced, these factors are handled through the addition of obstacle elements and position updates at each layer, making it unlikely that interaction growth will lead to a rapid increase in computational time. In this context, the proposed method improves RSU-based infrastructure applicability and can be regarded as an appropriate approach for generating reference paths for path negotiation in cooperative driving automation environments.

### 5.3. Limitations and Future Work

This study aimed to propose a path planning method for infrastructure-driven cooperative driving automation environments. Accordingly, there exist limitations that were not considered within the current scope, as well as aspects that may be extended in future work. First, the proposed method assumes full cooperation among CAVs and complete access to motion information in cooperative driving automation settings. Therefore, in mixed-traffic environments, or in cases where cooperative vehicles do not follow planned behaviors, or where the motion information of surrounding vehicles must be estimated through prediction, the validity of the planned paths may be reduced. This assumption was intentionally adopted in order to simplify the environment and analyze structural characteristics of the proposed approach. For the same reason, complex road geometries and mixed-traffic conditions were also restricted to maintain a simplified modeling environment.

In addition, the current framework updates trajectory decisions sequentially based on the most recently available information and does not explicitly perform re-planning under asynchronous or delayed inputs. Therefore, handling fully asynchronous data streams and designing dedicated re-planning strategies under severe communication degradation remain important directions for future research.

In the present study, interactions among multiple vehicles are modeled using a simple superposition (summation) of individual vehicle potential fields, which provides a computationally efficient baseline formulation for multi-vehicle interaction. Scenarios in which multiple vehicles simultaneously explore new paths may require multi-agent path generation techniques such as MAPF. Furthermore, scalability with respect to the number of vehicles and the spatial extent of the monitored road segment has not been explicitly addressed in this study. In large-scale traffic environments, strategies for limiting or sparsifying potential field contributions, as well as distributed or hierarchical planning approaches, may be required to ensure computational efficiency and stable performance. Furthermore, scalability with respect to the number of vehicles and the spatial extent of the monitored road segment has not been explicitly addressed in this study. In large-scale traffic environments, strategies for limiting or sparsifying potential field contributions, as well as distributed or hierarchical planning approaches, may be required to ensure computational efficiency and stable performance.

Furthermore, because this study was conducted from the viewpoint of a single ego vehicle, evaluating the impact of the proposed method from a system-level traffic perspective remains an important future research direction.

In summary, this study focused on method development and conducted experiments under a simplified modeling environment with controlled variables. Therefore, future work may extend the research by introducing more diverse environmental conditions and variables to validate the adaptability of the proposed method.

## Figures and Tables

**Table 1:** Simulation scenario parameters.

Category	Item	Value/Description
Road	Road type	Two-lane one way, straight section
	Road width	7 m
Ego vehicle	Initial position	(x, y) = (−75, 1, 75)
	Initial speed	16.66 m/s (60 km/h)
Leading vehicle	Initial position	(x, y) = (0, 1.75)
	Initial speed	11.11 m/s (40 km/h)
	Initial acceleration	$0.1\;m/s^{2}$
Goal point		(x, y) = (500, 1.75)

**Table 2:** Scenario evaluation indicators.

Category	Indicator	Description	Valuation
Safety	Minimum SDM (Safety Distance Margin)	Distance-based dimensionless metric considering vehicle size	A distance-based dimensionless metric that accounts for the vehicle body size, used to evaluate whether a safe separation distance from the preceding vehicle is maintained without collision or physical contact.
	Minimum TTC (time to collision	Time to collision (TTC) based on vehicle speed and longitudinal distance	A time-to-collision metric defined using the vehicle speed and longitudinal distance, providing a quantitative interpretation of collision risk by relating relative distance to motion dynamics.
Trajectory Quality	Maximum acceleration	Maximum rate of change in vehicle speed over time	The maximum rate of change in vehicle speed with respect to time, used to interpret the magnitude of dynamic state variations and their impact on ride comfort.
	Maximum yaw rate	Mean absolute rate of change in heading angle over time	The mean absolute value of the time derivative of the vehicle heading angle, serving as a supplementary measure to assess directional changes during trajectory execution.
Computational Efficiency	Computational time (s)	Computation time for path generation	The computation time required to generate a path, used to evaluate whether the proposed method can perform path planning within a short time frame.

**Table 3:** TTC thresholds reported in previous studies.

Study/Organization	Road Environment	Minimum (s)	Desirable (s)
[43]	Intersection	1.0	1.5
[44]	Low-Level Congestion Intersection	1.6	2.0
[45]	Intersection	1.0	2.0
[46]	Urban Tunnel	2.0	4.0

**Table 4:** Quantitative performance metrics under different time update intervals in GTS-PF.

Time Update Intervals (s)	Computational Efficiency	Safety		Trajectory Quality
	Computational Time (s)	Minimum SDM	Minimum TTC (s)	Maximum Acceleration (m/s^2^)
0.1	0.3300	1.12	8.849	19.290
0.2	0.1660	1.13	9.155	8.745
0.3	0.1099	1.13	9.480	5.830
0.4	0.0838	1.12	9.603	4.360
0.5	0.0683	1.19	9.855	3.447
0.6	0.0596	1.12	9.906	2.892
0.7	0.0494	1.19	10.190	2.485
0.8	0.0425	1.19	10.683	2.174
0.9	0.0411	1.18	11.445	1.931
1.0	0.0370	1.20	10.966	1.760

**Table 5:** Relative impact of latency on path planning performance (safety).

Category	Indicator	Threshold	RRT-STRF	GT-PF	GTS-PF
Safety	Minimum SDM	0.0	1.18	1.02	1.19
	Minimum TTC (s)	4.7	8.61	7.79	9.86

**Table 6:** Relative impact of latency on path planning performance (trajectory quality).

Category	Indicator	Threshold	RRT-STRF	GT-PF	GTS-PF
Trajectory quality	Maximum acceleration (m/s^2^)	4.00	85.50	209.90	3.45
	Maximum yaw rate (deg/s)	-	2.23	5.36	2.02

**Table 7:** Relative impact of latency on path planning performance (computational efficiency).

Category	Indicator	RRT-STRF	GT-PF	GTS-PF	Reduction Relative to GTS-PF (%)
Computational Efficiency	Computational Time (s)	8.2379	0.3591	0.0683	RRT-STRF: −99.17 GT-PF: −80.99

**Table 8:** Extension analysis scenario.

Configuration	Behavior	Figure	Scenario
Two-Vehicle Scenario	Car-following	Figure 11a and Figure 12	The ego vehicle starts with a sufficient separation distance while traveling at the same speed (60 km/h) as the preceding vehicle and maintains lane-center driving with a constant gap.
	Early overtaking	Figure 11b and Figure 13	The ego vehicle travels at a higher speed (80 km/h) than the preceding vehicle (40 km/h) and performs an early lane change to overtake before a potential collision occurs.
	Late overtaking	Figure 11c and Figure 14	The ego vehicle approaches the preceding vehicle (40 km/h) from a distance of 150 m while traveling at a relatively high speed (60 km/h), and performs a lane change to overtake after the separation distance becomes sufficiently small.
	CAV speed variation	Figure 15a and Figure 16	The preceding vehicle changes its speed from 40 km/h to 60 km/h, creating a situation in which lane-change and cut-in maneuvers for overtaking become potentially unsafe.
Multi-Vehicle Scenario	Merge	Figure 15b and Figure 17	The ego vehicle merges into another lane by inserting itself into a suitable gap between two vehicles traveling in that lane.
	Blocked lane return	Figure 15c and Figure 18	The ego vehicle does not perform a merge maneuver due to insufficient spacing between two vehicles in the adjacent lane.

**Table 9:** Safety indicators for sub-scenarios (scenario Figure 11 series).

Scenario	Description	Minimum TTC (s)	Minimum SDM
Figure 11a	Car-following	N/A	28.29
Figure 11b	Early overtaking	4.78	1.24
Figure 11c	Delayed overtaking	21.98	1.02

**Table 10:** Safety indicators for sub-scenarios (scenario 17 series).

Scenario	Description	Vehicle	Minimum TTC (s)	Minimum SDM
Figure 15a	Car-following	-	7.56	1.05
Figure 15b	Early overtaking	lead	0.96	1.41
		further lead	N/A	1.62
Figure 15c	Delayed overtaking	lead	7.50	1.42
		further lead	N/A	1.62

**Table 11:** Impact of sensing and communication uncertainty on path planning performance.

Category	Normal	Delay	Noise
Minimum TTC (s)	9.86	9.86	9.86
Minimum SDM	1.19	1.18	1.08

## Data Availability

The datasets generated and analyzed in this study are part of an ongoing research project and are currently being used for further publications. Therefore, the data are not publicly available at this time. However, the raw data supporting the conclusions of this article will be made available by the authors upon reasonable request.

## Abbreviations

The following abbreviations are used in this manuscript:

V2X	Vehicle-to-Everything
RSU	Road-Side Unit
APF	Artificial Potential Field
MPC	Model Predictive Control
PF	Potential Field
STRF	Spatial–Temporal Risk Field
SDM	Safety Distance Margin
TTC	Time To Collision
ADAS	Advanced Driver Assistance System
CAV	Connected Automated Vehicle
